# Overadjustment bias in systematic reviews and meta-analyses of socio-economic inequalities in health: a meta-research scoping review

**DOI:** 10.1093/ije/dyad177

**Published:** 2023-12-21

**Authors:** Anita van Zwieten, Jiahui Dai, Fiona M Blyth, Germaine Wong, Saman Khalatbari-Soltani

**Affiliations:** School of Public Health, Faculty of Medicine and Health, University of Sydney, Sydney, NSW, Australia; Centre for Kidney Research, Children’s Hospital at Westmead, Westmead, NSW, Australia; School of Public Health, Faculty of Medicine and Health, University of Sydney, Sydney, NSW, Australia; School of Public Health, Faculty of Medicine and Health, University of Sydney, Sydney, NSW, Australia; ARC Centre of Excellence in Population Ageing Research (CEPAR), University of Sydney, Sydney, NSW, Australia; School of Public Health, Faculty of Medicine and Health, University of Sydney, Sydney, NSW, Australia; Centre for Kidney Research, Children’s Hospital at Westmead, Westmead, NSW, Australia; Centre for Transplant and Renal Research, Westmead Hospital, Westmead, NSW, Australia; School of Public Health, Faculty of Medicine and Health, University of Sydney, Sydney, NSW, Australia; ARC Centre of Excellence in Population Ageing Research (CEPAR), University of Sydney, Sydney, NSW, Australia

**Keywords:** Epidemiology, overadjustment bias, confounding bias, systematic review, meta-analysis, health inequalities, health equity, health disparities, bias, meta-research

## Abstract

**Background:**

Overadjustment bias occurs when researchers adjust for an explanatory variable on the causal pathway from exposure to outcome, which leads to biased estimates of the causal effect of the exposure. This meta-research review aimed to examine how previous systematic reviews and meta-analyses of socio-economic inequalities in health have managed overadjustment bias.

**Methods:**

We searched Medline and Embase until 16 April 2021 for systematic reviews and meta-analyses of observational studies on associations between individual-level socio-economic position and health outcomes in any population. A set of criteria were developed to examine methodological approaches to overadjustment bias adopted by included reviews (rated Yes/No/Somewhat/Unclear).

**Results:**

Eighty-four reviews were eligible (47 systematic reviews, 37 meta-analyses). Regarding approaches to overadjustment, whereas 73% of the 84 reviews were rated as Yes for clearly defining exposures and outcomes, all other approaches were rated as Yes for <55% of reviews; for instance, 5% clearly defined confounders and mediators, 2% constructed causal diagrams and 35% reported adjusted variables for included studies. Whereas only 2% included overadjustment in risk of bias assessment, 54% included confounding. Of the 37 meta-analyses, 16% conducted sensitivity analyses related to overadjustment.

**Conclusions:**

Our findings suggest that overadjustment bias has received insufficient consideration in systematic reviews and meta-analyses of socio-economic inequalities in health. This is a critical issue given that overadjustment bias is likely to result in biased estimates of health inequalities and accurate estimates are needed to inform public health interventions. There is a need to highlight overadjustment bias in review guidelines.

Key Messages

- Overadjustment bias is an important issue in research on socio-economic inequalities in health as it may lead to biased estimates of inequalities, particularly in the context of systematic reviews, which are often a key source of evidence that informs policy and practice to address this pervasive social injustice.
- We found that approaches to address overadjustment bias were infrequently applied by included systematic reviews of socio-economic inequalities in health, suggesting that overadjustment bias has received insufficient consideration to date.
- These findings highlight the need to include overadjustment bias in guidelines for conducting and reporting systematic reviews, and to use recently developed risk of bias tools that consider overadjustment bias.

## Introduction

Inequalities in health between individuals of differing socio-economic position (SEP) are a pervasive social injustice. For instance, in a number of European countries, mortality rates are twice as high for individuals in the lowest compared with the highest educational groups and, across European countries, individuals with lower education levels have ∼1.5–4 times the risk of low self-rated health compared with those with higher levels of education.^1^ Therefore, high-quality evidence on health inequalities is essential to inform equity-focused interventions. However, socio-economic inequalities in health are inherently challenging to study given the complexity of the underlying causal mechanisms and because most evidence comes from observational studies, which are subject to multiple potential biases, notably confounding bias where a spurious association is introduced by a third variable that causes both the socio-economic exposure and health outcome.^2^ This highlights the importance of understanding and addressing potential biases inherent to observational studies. Managing bias is particularly important when synthesizing evidence across studies in systematic reviews because systematic reviews are often a key source of evidence that informs policy and practice.

Whereas existing guidelines for the conduct and reporting of systematic reviews and meta-analyses provide comprehensive guidance and tools to address confounding bias,^3–5^ overadjustment bias has historically been overlooked. Control for confounding is important to avoid the introduction of confounding bias, which may bias the estimated association in any direction, and is commonly achieved by adjusting for potential confounding variables in analytic models. However, variables for adjustment must be chosen carefully in order to avoid introducing other types of bias, particularly in health inequalities studies in which there are complex underlying pathways from SEP to health (including material, behavioural, psychosocial and biological mediators).^6^ Notably, overadjustment bias may occur when the total effect of an exposure is of interest and researchers mistakenly adjust for a mediator (or a descending proxy of a mediator) on a causal pathway from exposure to outcome.^7^ Adjustment for a mediator closes a causal pathway from the exposure to the outcome, thereby reducing the total causal effect that is estimated. This is likely to bias the estimated effect of the exposure on the outcome towards the null and so result in an underestimation of socio-economic inequalities in health.^7^ For example, a recent primary study of associations between educational attainment and clinical outcomes among people with chronic kidney disease showed that inclusion of potential mediators including health behaviours, disease progression and comorbidities resulted in substantial attenuation of educational inequalities in vascular events.^8^ If the mediator is also caused by other common causes of the outcome, adjustment for the mediator may also result in collider bias, which can bias the estimated effect of the exposure in any direction.^7^ It is therefore important that researchers carefully consider causal pathways when selecting variables for inclusion in statistical models to ensure that confounding is addressed while avoiding the introduction of bias through overadjustment.^9^

Understanding how systematic reviews have approached overadjustment is critical, as the presence of overadjustment bias in existing evidence may lead to systematic underestimation and other types of bias in the estimated effects of SEP on health outcomes.^10^ This scoping review therefore aimed to examine how previous systematic reviews of associations between SEP and health have managed the issue of overadjustment bias (both overall and in comparison with confounding bias).

## Methods

To conduct this meta-research review, we adopted a scoping review methodology following the PRISMA Extension for Scoping Reviews (PRISMA-ScR).^11^ A scoping review was considered appropriate given the breadth of the question and the focus on mapping the approaches taken by reviews in this area to address overadjustment bias. We did not publish a study protocol. Ethical approval was not required as this project was a review that did not involve any primary data.

### Literature search

Ovid Medline and Embase databases were searched from inception to 16 April 2021 for systematic reviews and meta-analyses on associations between individual-level SEP and health (i.e. socio-economic inequities/inequalities/disparities in health) limited to English-language results. The search strategy was developed collaboratively by the authors based on the review question and inclusion criteria and included medical subject headings (MeSH) and key words related to health inequalities (e.g. equity, inequality, social gradient, disparity) combined with those related to systematic reviews and meta-analyses. The full search strategy is available in the Supplementary material, Section A (available as Supplementary data at *IJE* online). Although reviews that focused on a single SEP domain were eligible for inclusion, terms for individual SEP domains (e.g. education, occupation, income) were not included in the search strategy for feasibility reasons, to keep the search size manageable. Because our scoping review was across health outcomes and regions, adding combined terms of health inequalities and individual SEP yielded a very large number of articles, which would have made the study infeasible. Additional reviews were identified by A.v.Z. and S.K.S. through expert knowledge of papers in the field.

### Inclusion and exclusion criteria

We included systematic reviews and meta-analyses of observational studies (cohort, cross-sectional and case–control designs) that assessed the association between any individual-level SEP measure (e.g. income, occupation, education, employment status, wealth, subjective social status, financial hardship) and any physical or mental health outcome (e.g. incidence/prevalence of a communicable or non-communicable disease, quality of life, self-rated health, mental health symptoms, mortality, sleep, biomarkers, overweight/obesity) in any human population. To be included, the review had to focus on total effects of SEP, be written in English and be published in a peer-reviewed journal. We restricted the search to reviews in English for feasibility reasons and to ensure that we could accurately interpret any discussion of overadjustment bias. We also restricted our search to peer-reviewed reviews given that our focus was on examining past and current practice in published reviews. We did not restrict our search on the basis of country of publication, publication date, length of follow-up or methodological quality. We did not limit the search to papers focused explicitly on causal inference, as existing work has highlighted that, despite using associational language in observational health research, researchers are often implicitly interested in the causal effect of an exposure on an outcome and interpret their findings accordingly with a causal intent.^12^^,^^13^

We excluded primary studies, systematic reviews of randomized–controlled trials, protocols of systematic reviews/meta-analyses and conference abstracts. Reviews that included some randomized–controlled trials alongside observational studies were eligible for inclusion. For protocols and conference abstracts, we searched for subsequent full-text publications for inclusion. Given that our focus was on the most commonly used individual-level SEP indicators, we excluded reviews in which the exposure was area-level SEP (e.g. area deprivation indices, income inequality measures) and less common proxy measures of SEP (e.g. living in public housing, having health insurance). We also excluded reviews in which the outcome was only related to health behaviours (e.g. diet, physical activity, alcohol consumption, smoking), health service use (e.g. hospital visits, general practitioner visits), access to healthcare (e.g. distance from hospital), uptake of healthcare (e.g. vaccination), adherence and knowledge or attitudes towards health/healthcare. These exclusions were made given the breadth and diversity of studies in the field of socio-economic inequalities in health and the need to ensure a sufficiently homogenous set of reviews for consideration of methodological approaches. Reviews that included some ineligible exposures/outcomes but also included some eligible exposures/outcomes were included, with a focus on the eligible exposures/outcomes in data extraction. Reviews focusing on mediation analysis of SEP or decomposition of indirect/direct effects of SEP were excluded, as were reviews that focused on methodological aspects or causal mechanisms of socio-economic inequalities in health rather than on describing total effects of SEP on health.

### Study selection

Search results from both databases were merged into Covidence Systematic Review Software^14^ and duplicates were removed. Titles and abstracts were independently screened by two reviewers (J.D. and L.Y.) using inclusion and exclusion criteria within Covidence. A second level of screening then took place in which the reviewers independently assessed the shortlisted full-text articles. Differences of opinion between reviewers were resolved through discussion with other authors (S.K.S. and A.v.Z.). A second set of reviewers (S.K.S. and A.v.Z.) checked the results of the full-text screening.

### Data extraction

Data were extracted from eligible reviews using a standardized data extraction form in Microsoft Excel developed by the authors for this review. Details of the data extraction are included in the Supplementary material, Section B (available as Supplementary data at *IJE* online).

### Approaches to overadjustment bias

For the purposes of this review, we developed a set of criteria to examine approaches to overadjustment across included reviews; there was not an existing set of criteria for this purpose to our knowledge. These criteria were developed with reference to theoretical/methodological literature on overadjustment^7^ and previous primary studies, systematic reviews and meta-analyses in this area with explicit consideration of overadjustment.^8^^,^^10^^,^^15^ Of note, these criteria were focused on how the authors of the systematic reviews managed overadjustment bias and not on how overadjustment had been managed in the primary studies included in the review. Regarding the approaches to addressing overadjustment bias, we extracted information on whether each included review: (i) had clearly defined exposure(s) and outcome(s), (ii) had clearly defined relevant confounder(s) and mediator(s), (iii) presented causal diagrams [e.g. directed acyclic graphs (DAGs)], (iv) included overadjustment in risk of bias (ROB) assessment, (v) included confounding in ROB assessment, (vi) conducted sensitivity analyses related to overadjustment (only for meta-analyses), (vii) reported which variables were adjusted for in each included study, (viii) prioritized results from models with proper adjustment, (ix) prioritized results from minimally adjusted models, (x) presented results from models with different levels of adjustment for comparison, (xi) had discussion of overadjustment in the text and/or (xii) used other approaches to manage overadjustment. Criteria were rated as Yes/No/Somewhat/Unclear, with some criteria also having a not applicable (n/a) option where relevant (Criterion 6 had the option of n/a: no meta-analysis and Criteria 4 and 5 had the option of n/a: no ROB assessment). Further details on the assessment of each criterion are provided in the Supplementary material, Section C (available as Supplementary data at *IJE* online).

### Critical appraisal

We carried out critical appraisal of the quality of included reviews to provide context on the overall quality of the reviews alongside their management of overadjustment bias. Two reviewers (S.K.S. and A.v.Z.) critically appraised the included systematic reviews using a modified 16-item AMSTAR-2 checklist^16^ (A MeaSurement Tool to Assess systematic Reviews). Reviews were divided between the two authors for appraisal. The checklist was tailored with minor amendments to suit the review, as detailed in the Supplementary material, Section D (available as Supplementary data at *IJE* online).

### Synthesis of results

We summarized review characteristics, approaches to overadjustment bias, and confounding, and critical appraisal results descriptively, reporting numbers and percentages across categories.

## Results

### Study selection

**Figure 1.**
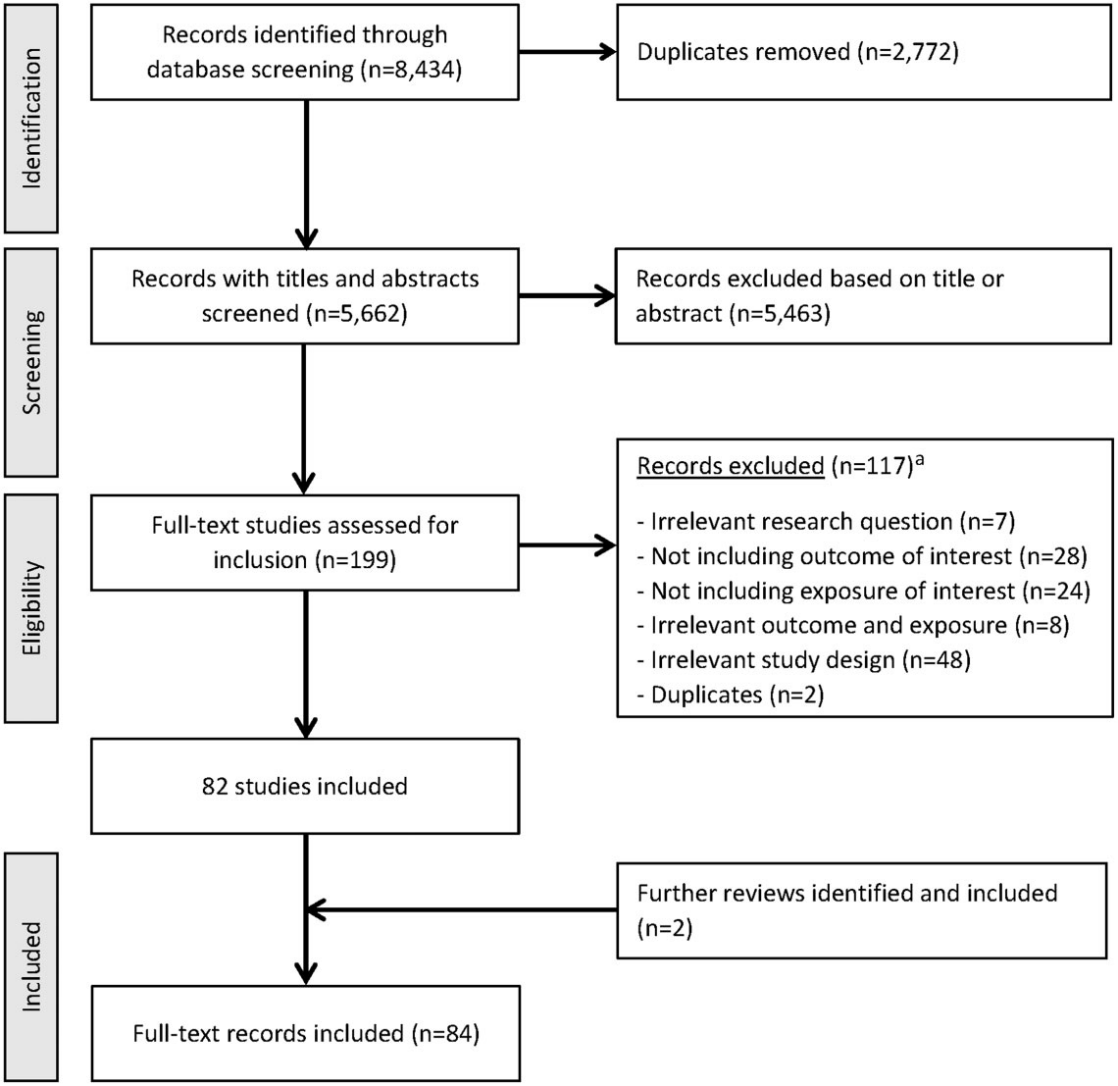
Study selection flow diagram. ^a^See Supplementary Table S1 (available as Supplementary data at *IJE* online) for list of articles excluded during full-text screening

The study selection flowchart is presented in Figure 1. A total of 8434 articles were identified and, after excluding duplicates and those not meeting eligibility criteria based on title and abstract screening, 199 articles progressed to full-text screening. Of these, 82 articles were considered eligible. Supplementary Table S1 (available as Supplementary data at *IJE* online) shows the list of excluded articles at the full-text screening step with reasons (*n* = 117). Two additional reviews were included through discussion with experts, taking the total number of included reviews to 84.^10^^,^^15^^,^^17–98^

### Characteristics of included reviews

The characteristics of each study are presented in Supplementary Table S2 (available as Supplementary data at *IJE* online) and characteristics are summarized across reviews in Table 1. Of 84 included reviews, 47 were systematic reviews without meta-analysis and 37 included meta-analysis. Most reviews included longitudinal (*n* = 50) and cross-sectional (*n* = 46) studies, and some reviews also considered case–control (*n* = 23) study designs. Almost all (*n* = 75) of the included reviews were published after 2010. Most reviews had some type of population restrictions, particularly country or age restrictions, and six reviews only included female participants. Education (*n* = 57), occupation (*n* = 49) and income (*n* = 49) were the most common SEP indicators considered, followed by parental (*n* = 29), composite (*n* = 14) or subjective (*n* = 5) SEP measures. Health outcomes varied across included reviews, with the most common being cardiovascular disease-related, cancer-related and weight-related (*n* = 9 each) outcomes, followed by self-rated health and quality of life (*n* = 8), diabetes-related (*n* = 7) and oral health-related (*n* = 6) outcomes.

**Table 1.** Overall characteristics of included systematic reviews and meta-analyses (*n *=* *84)

Characteristic	Number of reviews (%)
Type of review [*n* (%)]	
Systematic review only	47 (56)
Systematic review and meta-analysis	37 (44)
Number of included studies in review (range)	7–183
Year of publication [*n* (%)]	
2000–09	9 (11)
2010 onwards	75 (89)
Population of interest [*n* (%)]	
Adults only (aged >18 years)	27 (32)
Children only (aged <21 years)	14 (17)
Both adults and children	10 (12)
Not stated	33 (39)
Region/country [*n* (%)]	
No restrictions	27 (32)
High-income countries	17 (20)
Low- and middle-income countries	9 (11)
Mixed	12 (14)
Not stated	18 (22)
Unclear	1 (1)
Type of studies included in review^a^	
Longitudinal	50
Cross-sectional	46
Case–control	23
Other	11
Not stated	22
Gender-related population restrictions (female only)^b^	6
Socio-economic position indicator(s)^a^	
Education	57
Occupation/employment status	49
Income	49
Subjective socio-economic position	5
Parental socio-economic position	29
Composite socio-economic position	14
Other^c^	25
Unclear definition	2
Outcome(s)^a^	
Cardiovascular disease-related outcomes	9
Cancer-related outcomes	9
Weight-related outcomes	9
Self-rated health; proxy-rated health; quality of life	8
Diabetes-related outcomes	7
Oral health-related outcomes	6
Mortality	4
Infection (e.g. gastrointestinal, malaria, HIV, Chlamydia)	4
Other^d^	31

a For these characteristics, reviews may appear in more than one category, so the total may be >84 and percentages are therefore not presented.

b All reviews that included gender-related restrictions limited the population to females.

c Other socio-economic indicators included poverty, deprivation, housing condition, financial hardship, housing tenure and car ownership.

d Other outcomes included maternal-related outcomes, gout, chronic kidney disease, ageing, injury, visual impairment, neglected tropical diseases, inflammatory markers, chronic obstructive pulmonary disease, sleep disorders, dementia, epilepsy and depression.

### Critical appraisal of included reviews using AMSTAR 2

**Figure 2.**
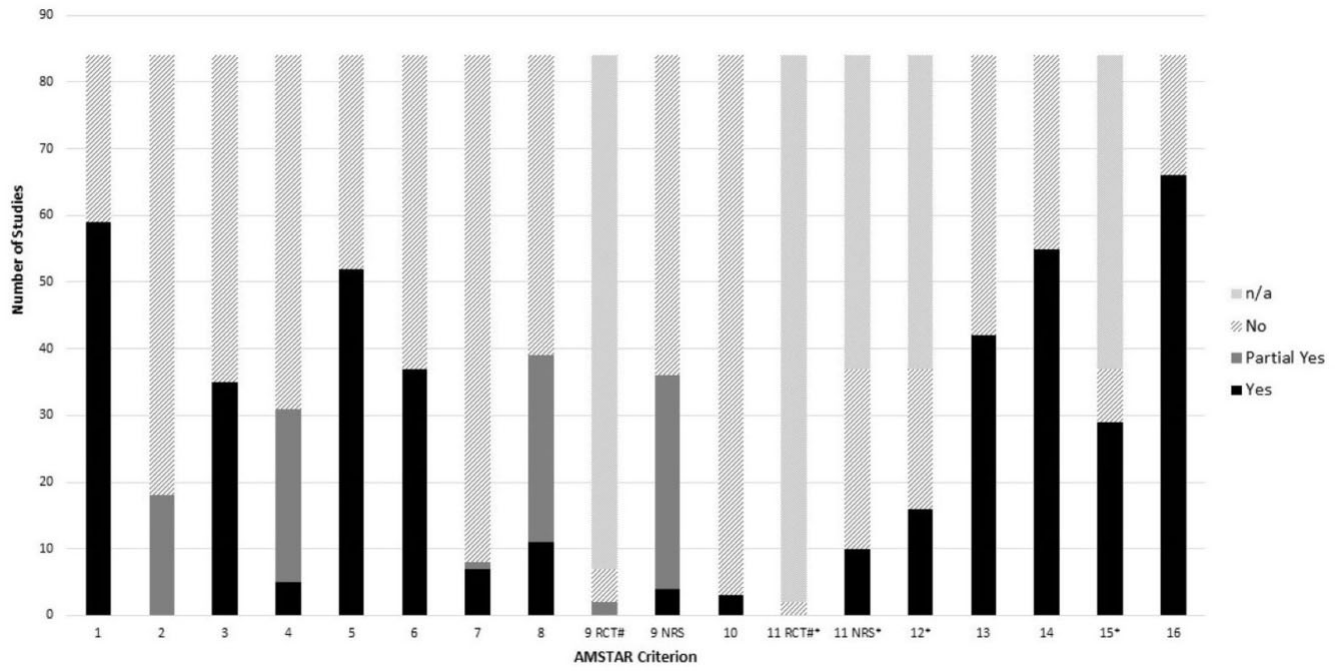
Number of reviews receiving each AMSTAR-2 rating in critical appraisal of included reviews (*n* = 84). ^a^Only applied to meta-analyses. ^#^Only applied to reviews including RCTs. AMSTAR 2 items are as follows: 1. Defined PICO question (Population, Intervention, Comparator, Outcome). 2. A priori methods in protocol. 3. Justified inclusion of study designs. 4. Comprehensive literature search. 5. Duplicate study selection. 6. Duplicate data extraction. 7. Provided list of excluded studies and justification. 8. Detailed description of included studies. 9. Satisfactory ROB assessment. 10. Reported funding sources for included studies. 11. Appropriate methods for meta-analysis. 12. Assessed impact of ROB on meta-analysis results. 13. Accounted for ROB when interpreting results. 14. Explained and discussed any heterogeneity. 15. Assessed publication bias. 16. Review authors reported potential conflicts of interest including funding. RCT, randomized–controlled trial; NRS, non-randomized study; ROB, risk of bias; AMSTAR, A MeaSurement Tool to Assess systematic Reviews

Results of the critical appraisal of each included review using AMSTAR 2 are presented in Supplementary Table S3 (available as Supplementary data at *IJE* online) and are summarized in Figure 2 across reviews as the number in each category for each criterion. Of note, the percentages of eligible reviews with a ‘Yes’ for satisfactory ROB assessment including confounding, selection and measurement biases plus selective reporting (Domain 9 for non-randomized studies) was 5% (*n* = 4), whereas consideration of ROB in individual primary studies when interpreting results (Domain 13) was met by 50% of reviews (*n* = 42). Of the other domains, the percentage of eligible reviews not complying (those receiving a ‘No’ classification out of those for which this criterion was relevant) was highest for Domains 10 (funding source disclosed: *n* = 81, 96%), 7 (list of excluded studies and justification: *n* = 76, 90%), 2 (a priori methods: *n* = 66, 79%) and 11 non-randomized studies (appropriate methods for meta-analysis: *n* = 27, 73%). The percentage of eligible reviews complying (those receiving a ‘Yes’ classification out of those for which this criterion was relevant) was highest for Domains 15 (publication bias: *n* = 29, 78%), 16 (conflicts of interest: *n* = 66, 79%) and 1 (inclusion of Population, Intervention, Comparator, Outcome in research question and inclusion criteria: *n* = 59, 70%). Domain 9 for randomized–controlled trials (RCTs) (ROB assessment satisfactory) and Domain 11 for RCTs (appropriate methods for meta-analysis) were relevant to very few included reviews, given that our scoping review focused on the reviewing of observational studies.

### Approaches to managing overadjustment bias

Supplementary Table S4 (available as Supplementary data at *IJE* online) shows the approaches towards managing overadjustment bias applied by each included review, summarized across reviews (*n* = 84) in Table 2. Most reviews had clearly defined both their exposure(s) and outcome(s) (*n* = 61, 73%), with 18 (21%) somewhat applying this approach. In contrast, only 5% (*n* = 4) of reviews had clearly defined both their confounder(s) and mediator(s), with 35% (*n* = 29) somewhat applying this approach. Of the 29 receiving a somewhat rating, 41% were rated as somewhat or not clear for mediators but yes for confounders; 10% were rated as not clear for confounders but yes for mediators; and 31% were rated as not clear or somewhat clear for both mediators and confounders. Only 2% (*n* = 2) of reviews constructed causal diagrams, with 1% (*n* = 1) somewhat applying this approach. This one study provided a conceptual model and not a causal diagram per se. Similarly, overadjustment was included in formal ROB assessment by only 2% of reviews (*n* = 2) and somewhat included by 1% (*n* = 1). For the remainder of reviews, they either did not include overadjustment (*n* = 45, 54%), it was unclear (*n* = 11, 13%) or they did not report a formal ROB assessment (*n* = 25, 30%). In contrast, confounding was included in formal ROB assessment by 54% (*n* = 45) of reviews and somewhat included by 2% (*n* = 2), with only 2% (*n* = 2) of reviews not including it in their ROB assessment and inclusion being unclear for 12% (*n* = 10). Most reviews either reported which variables were adjusted for in each included study (*n* = 29, 35%) or somewhat applied this approach (*n* = 14, 17%), with this criterion being unclear for 1% of reviews (*n* = 1). Authors prioritized results from models with proper adjustment in 13% of reviews (*n* = 11), with 1% (*n* = 1) somewhat adopting this approach and 6% (*n* = 5) being unclear. Only 5% of reviews prioritized results from minimally adjusted models (*n* = 4), with this criterion being unclear for 6% (*n* = 5) of reviews. A moderate number of reviews presented results with different levels of adjustment for comparison, with 27% (*n* = 23) applying and 15% (*n* = 13) somewhat applying this approach. Overadjustment was discussed in the text of 27% of reviews (*n* = 23) and somewhat discussed by 2% (*n* = 2). Other approaches related to overadjustment were applied by 10% (*n* = 8) of reviews, most of which related to reporting the results of mediation analyses for included studies (Supplementary Table S4, available as Supplementary data at *IJE* online). Of the meta-analyses (*n* = 37), 16% (*n* = 6) conducted sensitivity analyses related to overadjustment, with 73% not applying (*n* = 27) and 11% (*n* = 4) somewhat applying this approach (Table 2).

**Table 2.** Number (%) of reviews applying different approaches that address overadjustment bias

Approach	Number of reviews (%)
Had clearly defined exposure(s) and outcome(s)	
Yes	61 (73)
No	5 (6)
Somewhat	18 (21)
Unclear	0
Had clearly defined confounder(s) and mediator(s)	
Yes	4 (5)
No	51 (61)
Somewhat	29 (35)
Unclear	0
Constructed causal diagrams	
Yes	2 (2)
No	81 (96)
Somewhat	1 (1)
Unclear	0
Included overadjustment in risk of bias assessment	
Yes	2 (2)
No	45 (54)
Somewhat	1 (1)
Unclear	11 (13)
n/a—no formal risk of bias	25 (30)
Included confounding in risk of bias assessment	
Yes	45 (54)
No	2 (2)
Somewhat	2 (2)
Unclear	10 (12)
n/a—no formal risk of bias	25 (30)
Reported variables adjusted for in each included study	
Yes	29 (35)
No	40 (48)
Somewhat	14 (17)
Unclear	1 (1)
Prioritized results from models with proper adjustment	
Yes	11 (13)
No	67 (80)
Somewhat	1 (1)
Unclear	5 (6)
Prioritized results from minimally adjusted models	
Yes	4 (5)
No	75 (89)
Somewhat	0
Unclear	5 (6)
Presented results with different levels of adjustment for comparison	
Yes	23 (27)
No	48 (57)
Somewhat	13 (15)
Unclear	0
Discussed overadjustment in the text	
Yes	23 (27)
No	59 (70)
Somewhat	2 (2)
Unclear	0
Other approaches related to overadjustment	
Yes	8 (10)
No	76 (90)
Conducted sensitivity analyses related to overadjustment^a^	
Yes	6 (16)
No	27 (73)
Somewhat	4 (11)
Unclear	0

Criteria are outlined in detail in the Supplementary material, Section B (available as Supplementary data at *IJE* online). The total number of reviews included in this scoping review is 84 (47 systematic review only, 37 systematic review with meta-analysis).

a This criterion only applies to meta-analyses, so the denominator is 37. For all other criteria, the denominator is 84.

The total number of approaches to overadjustment applied across reviews is shown in Supplementary Figure S1a (available as Supplementary data at *IJE* online) for systematic reviews (*n* = 47) and Supplementary Figure S1b (available as Supplementary data at *IJE* online) for meta-analyses (*n* = 37). For systematic reviews, the number of approaches applied ranged from 0 to 6 (out of 11 possible approaches), with the most common being 1 approach (23%), closely followed by 2 (21%) and 3 and 0 (17% each) approaches. For meta-analyses, the number of approaches applied ranged from 0 to 9 (out of 12 possible approaches), with the most common being 3 (27%), closely followed by 2 (24%) approaches.

## Discussion

Overall, the findings of this scoping review suggest that overadjustment bias has received insufficient consideration in systematic reviews and meta-analyses of socio-economic inequalities in health to date. Across the 84 included reviews, approaches to address overadjustment bias were not regularly applied. The majority of systematic reviews applied fewer than three approaches, whereas the majority of meta-analyses applied fewer than four approaches. Whereas most (73%) reviews clearly defined their exposure(s) and outcome(s), all other approaches were applied in <55% of reviews. In particular, <5% of included reviews clearly defined confounders and mediators, constructed causal diagrams or conducted a ROB assessment that included overadjustment. In contrast, 54% of reviews conducted a ROB assessment with consideration of confounding. Given that 90% of included reviews were published after 2010 and therefore in the modern era of systematic reviews, it is concerning that ∼30% did not report a formal ROB assessment at all.

Potential risks of both confounding and overadjustment are important to consider in systematic reviews and meta-analyses of the impact of SEP on health. Overall, very few reviews clearly defined both confounders and mediators, and, among reviews that somewhat met this criterion, there were more likely to be issues with defining mediators than confounders. Of note, only 2% of included reviews explicitly evaluated overadjustment bias in ROB assessment, whereas assessment of confounding bias in ROB assessments was substantially higher, at 54%. This is not surprising given that the majority of guidelines and ROB tools for systematic reviews over the preceding decades have included explicit consideration of confounding bias. Of note, a 2007 review of ROB and quality assessment tools found that 78% addressed control of confounding (consideration of overadjustment bias was not assessed).^99^ On the other hand, overadjustment bias has historically been overlooked in guidance and tools for reviews, and has only been explicitly included in a few of the most recent ROB tools (e.g. ROBINS-E^100^ and ROBINS-I^101^). Our results are therefore likely to reflect a sparsity of recommendations to guide reviewers on overadjustment bias and accordingly a lack of awareness of and knowledge about overadjustment bias in the research community.

Given the potential impacts of overadjustment bias on estimated associations between SEP and health—notably the potential for underestimation of socio-economic inequalities in health—there is a critical need for further practical guidance and support for systematic reviewers in this area. Such guidance is likely to have broader benefits given that many of the approaches we assessed could also have other potential cross-benefits for enhancing the robustness of review findings. For example, clearly defining exposures, outcomes, confounders and mediators, and constructing causal diagrams that encode these assumptions, is likely to assist in managing confounding bias, which may in turn lead to more robust conclusions. Similarly, reporting the variables adjusted for in each study assists in considering confounding and has implications for the comparability of associations across reviews with different levels of adjustment. Adoption of these approaches is also likely to help readers to better interpret the results of reviews through detailed information on the theoretical models and potential mechanisms through which SEP influences health, which can in turn can inform potential interventions.

**Figure 3.**
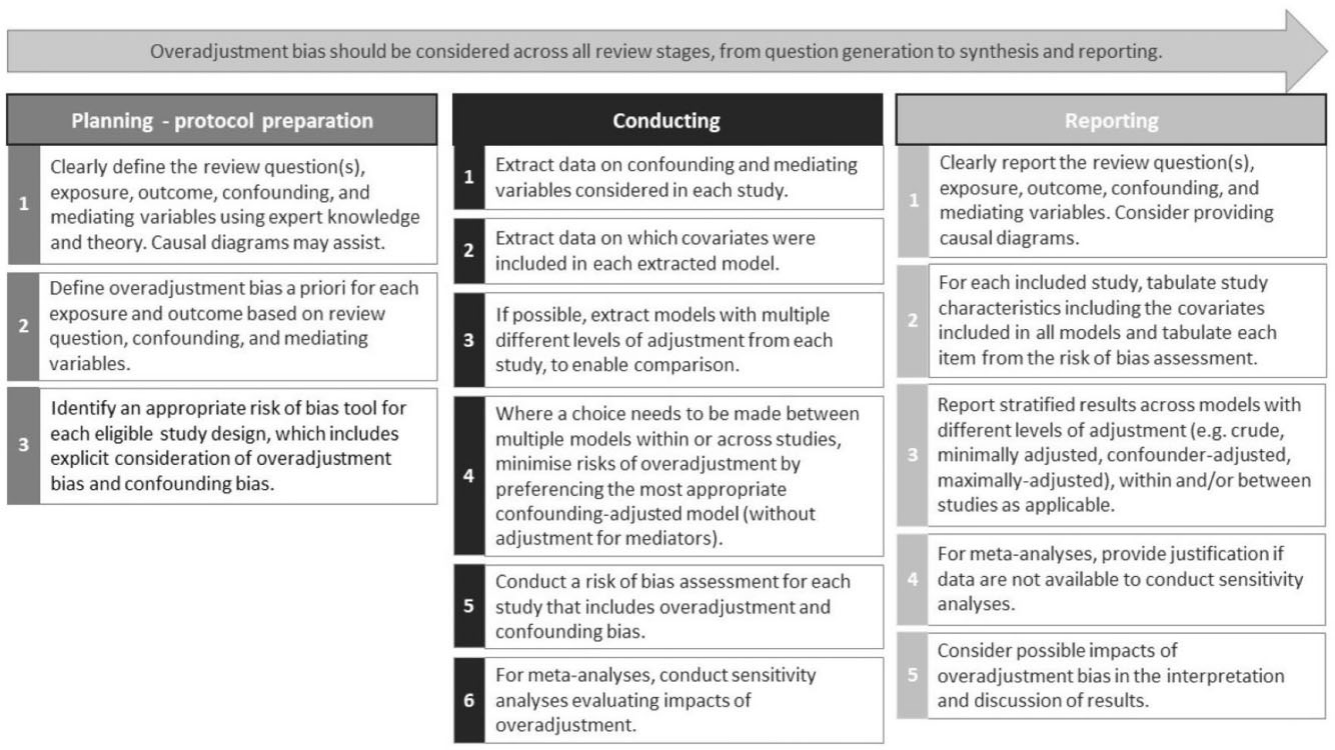
Summary of considerations for management of overadjustment bias in systematic reviews and meta-analyses

The lack of adequate management of overadjustment bias in the current body of evidence identified in the present review, in conjunction with the importance of accurate estimates of socio-economic inequalities in health for health policy and practice, warrant an increased emphasis on the importance of overadjustment bias in conduct and reporting guidelines and ROB assessment tools for systematic reviews in this area. Overadjustment bias should be considered and addressed across all stages of systematic reviews, from planning and protocol development to review conduct and reporting. The development of future tools and guidelines through consensus processes is an important next step. In the meantime, as a starting point, we provide here a summary of considerations for researchers when seeking to address overadjustment bias in systematic reviews of socio-economic inequalities in health in each review stage (Figure 3). We acknowledge that the scope of these considerations extends only to management of overadjustment bias by systematic reviewers, who cannot modify the extent of overadjustment bias in the primary evidence itself. There is, therefore, a need for concurrent action to reduce overadjustment bias in primary studies, as discussed elsewhere.^9^

Ours is the first to review how overadjustment bias has been managed in previous systematic reviews and meta-analyses of socio-economic inequalities in health. We applied rigorous methods including a systematic search across two key health databases and double review of reviewer decisions (where feasible). Nonetheless, there are some limitations. In order to ensure a manageable number of results for screening, we limited our search terms to those related to equity (without terms related to individual domains of SEP), meaning that some reviews may have been missed. We also excluded reviews that only focused on area-level SEP and less commonly used proxy measures of SEP. This was appropriate given that this study was a scoping review that aimed to provide an overview of approaches to overadjustment across a set of systematic reviews, rather than a systematic review aiming to synthesize results from all reviews. Whereas we limited our search to two databases for feasibility reasons, these were two major databases in public health and this allowed us to screen all results rather than selecting only a subset. Given the novelty of this topic, the coding system for examining approaches to overadjustment was developed as a preliminary version for the purposes of this scoping review. Future steps of this project will involve consultation, consensus-building and piloting processes to develop standardized tools, guidelines and criteria for broader use. Future work also needs to examine the impact of adopting such approaches on robustness of the review results. Coding was complex, with some ambiguity in certain domains (e.g. definitions of ‘proper adjustment’), some overlap across domains (e.g. sensitivity analyses for overadjustment and reporting of models with varying levels of adjustment) and some domains being mutually exclusive (e.g. prioritizing results with proper adjustment vs minimally adjusted results). There were also some methodological issues that emerged during the review process (e.g. reviewers suggesting that mediators should be adjusted for or incorrectly identifying variables as confounders when they were mediators), which should be in future studies. Our review relied on what the authors reported; we acknowledge that the authors might have considered some approaches to address overadjustment and/or confounding bias, but omitted details in their papers due to word limits. Given the scoping nature of our review, included reviews were highly heterogeneous in their aims and methods, and future meta-research could consider whether approaches to overadjustment differ across these parameters. For example, consideration of overadjustment bias and confounding is most relevant to reviews with a focus on causal inference. However, we did not limit our scoping review solely to reviews with an explicitly stated causal intent, as implicit investigation of causal effects is common practice in epidemiological research. We were not able to account for this in our review as we did not have sufficient information to evaluate the causal intent of included reviews. There were also some challenges with applying the AMSTAR 2 criteria to the included reviews, given that they focus on systematic reviews of interventions whereas we were focused on reviews of observational studies of SEP and health.

In conclusion, whereas confounding bias is increasingly being considered in systematic reviews of health inequalities, overadjustment bias has often been overlooked. This is a critical issue given the importance of having accurate estimates of socio-economic inequalities in health to inform policy and practice. To avoid overadjustment bias in future systematic reviews and to have more robust results, there is a need to highlight overadjustment bias in systematic review guidelines for conduct and reporting, and to use recent ROB tools that include overadjustment bias for systematic reviews.

## Ethics approval

Ethics approval was not required for this project as it is a scoping review that does not involve primary data.

## Supplementary Material

dyad177_Supplementary_Data

## Data Availability

Data from this review will be shared on reasonable request.
