# Supplementary material for: Overadjustment bias in systematic reviews and meta-analyses of socio-economic inequalities in health: a meta-research scoping review
Source: Int J Epidemiol. 2023 Dec 21;53(1):dyad177. doi: 10.1093/ije/dyad177 (PMC10859162; doi:10.1093/ije/dyad177)
Supplement: dyad177_Supplementary_Data [file dyad177_supplementary_data.docx]

**Supplementary Material**

**Contents**

[Section A. Search strategies 2](#_Toc152152646)

[Section B. Details of data extraction 3](#_Toc152152647)

[Section C. Criteria considered when evaluating approaches towards overadjustment in included systematic reviews and meta-analyses 4](#_Toc152152648)

[Section D. Details of AMSTAR-2 assessment 6](#_Toc152152649)

[Table S1. Excluded articles with main reason for exclusion (n=117) 7](#_Toc152152650)

[Table S2. Characteristics of included systematic reviews and meta-analyses 8](#_Toc152152651)

[Table S3. Critical appraisal for included studies using AMSTAR 2 21](#_Toc152152652)

[Table S4. Approaches towards addressing overadjustment (OA) bias for included studies 24](#_Toc152152653)

[Figure S1. Number of approaches to overadjustment applied by included studies 30](#_Toc152152654)

## Section A. Search strategies

*MEDLINE*

| **Block 1: equity terms** | equit*.tw or inequit*.tw or disparit*.tw or equalit*.tw or inequalit*.tw or social gradient.tw or Health Status Disparities/ or Health Equity/ |
| --- | --- |
| **Block 2: systematic review and meta-analysis terms** | systematic review.tw or meta-analy*.tw or meta analy*.tw or metaanaly*.tw or Systematic Review/ or Meta-Analysis/ |

**MEDLINE search strategy**: Block 1 **AND** Block 2 (limited to English language)

*EMBASE*

| **Block 1: equity terms** | equit*.tw or inequit*.tw or disparit*.tw or equalit*.tw or inequalit*.tw or social gradient.tw or Health disparity/ or health equity/ |
| --- | --- |
| **Block 2: systematic review and meta-analysis terms** | systematic review.tw or meta-analy*.tw or meta analy*.tw or metaanaly*.tw or Systematic Review/ or Meta-Analysis/ |

**EMBASE search strategy**: Block 1 **AND** Block 2 (limited to English language)

## Section B. Details of data extraction

For the majority of included reviews, they were divided between two authors for extraction (JD and LY) and then two other reviewers divided the studies and checked the data extraction (SKS and AvZ). There were a small number of additional reviews where only one of the reviewers (SKS or AvZ) extracted data. Information extracted included the review citation, aims, inclusion and exclusion criteria, whether the review included meta-analysis, number and types of studies included, and population and country restrictions. We also extracted details of the exposure type and timing, outcome type and timing, main conclusion of the review, approaches to overadjustment as outlined below, and for meta-analyses the sensitivity analyses conducted and summary of results.

## Section C. Criteria considered when evaluating approaches towards overadjustment in included systematic reviews and meta-analyses

1. **Clearly defined exposure(s) and outcome(s) –** did the authors clearly define the exposure(s) and outcome(s) for the causal question their review was addressing? *Options: Yes/No/Somewhat/Unclear^#^. If both yes: Yes. If both no: No. Otherwise (e.g. one Yes and the other No, one Yes and the other Somewhat, both Somewhat): Somewhat^.*
2. **Clearly defined confounders and mediators –** did the authors clearly define relevant confounders and mediators for the causal question their review was addressing? *Options: Yes/No/Somewhat/Unclear^#^. If both yes: Yes. If both no: No. Otherwise (e.g. one Yes and the other No, one Yes and the other Somewhat, both Somewhat): Somewhat.*
3. **Causal diagrams –** did the authors include a causal diagram (e.g. a directed acyclic graph) to illustrate the causal model underpinning their review? *Options: Yes/No/Somewhat^*^/Unclear.*
4. **Included overadjustment in risk of bias (ROB) assessment –** did the authors evaluate overadjustment bias as part of their ROB assessment/quality/critical appraisal for included studies? *Options: Yes/No/Somewhat^*^/Unclear^#^/n/a – no ROB. If no systematic risk of bias assessment was reported: n/a – no ROB.*
5. **Included confounding in ROB assessment –** did the authors evaluate confounding bias as part of their ROB assessment/quality/critical appraisal for included studies? *Options: Yes/No/Somewhat^*^/Unclear^#^/n/a – no ROB. If no systematic risk of bias assessment was reported: n/a – no ROB.*
6. **Sensitivity analyses related to overadjustment (only for meta-analyses) –** if it was a meta-analysis, did the authors conduct any sensitivity analyses that investigated or accounted for overadjustment bias? For example, meta-regressions examining the impact of adjustment for certain variables or comparing crude and adjusted results. *Options: Yes/No/Somewhat^*^/Unclear^#^/n/a. If not a meta-analysis: n/a.*
7. **Reported variables adjusted for in each included study –** did the authors report the variables that were adjusted for in the model(s) from each included study? *Options: Yes/No/Somewhat^*^/Unclear^#^.*
8. **Prioritised results from models with proper adjustment –** where multiple sets of results were available within an included study, or a choice needed to be made between different studies for inclusion, did the authors prioritise those with “proper adjustment” (where “proper adjustment” was as defined by the authors)? This included prioritising models adjusted for key confounders and/or those that were not adjusted for potential mediators. It did not include selecting maximally adjusted models. *Options: Yes/No/Somewhat^*^/Unclear^#^.*
9. **Prioritised results from minimally adjusted models –** where multiple sets of results were available within an included study, or a choice needed to be made between different studies for inclusion, did the authors prioritise those with minimal adjustment? *Options: Yes/No/Somewhat^*^/Unclear^#^.*
10. **Presented results with different levels of adjustment for comparison –** did the authors present results from models with different levels of adjustment for comparison? This could be within studies (extracted results with different levels of adjustment from each study where available, and presented for comparison) or across studies (compared results with different levels of adjustment across studies). Approaches could include: stratification or subgroup analyses comparing adjusted and unadjusted results in meta-analyses, stratified tabulation of adjusted and unadjusted results, comparison of adjusted and unadjusted results in the text. *Options: Yes/No/Somewhat^*^/Unclear^#^.*
11. **Had discussion in the text –** did the authors comment on issues related to overadjustment bias in the text? This could include: as part of the study background, when justifying or describing the methods, when describing and interpreting the results, and/or when discussing the limitations, implications and future directions of the review. *Options: Yes/No/Somewhat^*^/Unclear^#^.*
12. **Other approaches related to overadjustment:** did the authors take another approach to manage overadjustment bias not listed above? *Options: Yes/None. If yes, describe in text.*

*Notes*:

*For those criteria with a *Somewhat* response option (aside from 1 and 2 where *Somewhat* is defined above), this was used qualitatively as an intermediate option for reviews that did not fully satisfy the *Yes* response for that criterion but did partially satisfy the criterion. #For those criteria with an *Unclear* option, this was used where it was not possible to judge whether the criterion had been met (e.g. due to lack of information).

^ For clearly defined exposure and outcome, we classified reviews as “Yes” when the authors explicitly explained both the exposure and outcome of interest, “Somewhat” when only one of these was clearly defined or both were somewhat clearly defined, and “No” when no clear explanation/definition was provided for both exposure and outcome. Common situations where a “Somewhat” or “No” rating was given included those where a broad construct was named as exposure or outcome without further specification (e.g., early-life conditions, social determinants of health, socioeconomic position or socioeconomic status (for exposure), health (for outcome) listed without further clarification of which levels/domains were included from this construct or how it was defined).

## Section D. Details of AMSTAR-2 assessment

We made the following amendments to the AMSTAR-2 checklist to suit the review: for question 2 (a priori methods) the highest possible score was ‘Partial Yes’ as reviewers did not compare the protocol to the review to satisfy the ‘Yes’ criterion (due to time constraints), for question 3 (explanation of study selection) “explanation for including only non-randomized studies of interventions (NRSI)” was modified to “explanation for including only NRSI or specific designs” (because most studies in this field are observational so the specific designs are a more relevant distinction) and for question 4 (comprehensive literature search) the requirement to search trial/study registries for a ‘Yes’ score was removed (as our review focused on systematic reviews of observational studies not trials) and the requirement to search for grey literature for a ‘Yes’ score was removed (as grey literature was not considered critical for this review topic).

## Table S1. Excluded articles with main reason for exclusion (n=117)

| **Reason for exclusion** | **N of articles** | **References** |
| --- | --- | --- |
| Irrelevant research question | 7 | ^1-7^ |
| Not including outcome of interest | 28 | ^8-35^ |
| Not including exposure of interest | 24 | ^36-59^ |
| Irrelevant outcome and exposure | 8 | ^60-67^ |
| Irrelevant study design | 48 | ^68-115^ |
| Duplicates | 2 | ^116, 117^ |

## Table S2. Characteristics of included systematic reviews and meta-analyses

| **First Author (Year)** | **Citation** | **Aim of review** | **Systematic review with meta-analysis** | **Total N of studies included** | **Study types**  **(N of each)** | **Population** | **Exposure  Variables** | **Outcome Variables** |
| --- | --- | --- | --- | --- | --- | --- | --- | --- |
| Adams (2018) | ^118^ | To explore the relationship between SES and gastrointestinal infections. | Yes | 102 | Longitudinal (n=16)  Cross-sectional (n=18)  Case-control (n=25)  Ecological (n=43) | OECD countries  No age restrictions | Education  Occupation  Income | Incidence/prevalence  of gastrointestinal infection |
| Adegbosin (2019) | ^119^ | To meta-analyse existing literature on association between three major equity stratifiers and a selection of Reproductive, Maternal, Newborn and Child Health indicators. | Yes | 28 | Longitudinal (n=1)  Cross-sectional (n=26)  Cluster RCT (n=1) | Low- and middle-income countries  Maternal, newborn, and child | Education  Income | Reproductive, Maternal, Newborn and Child Health indicators (e.g., stunting in children under five, Maternal Mortality Rate, unmet need for contraception) |
| Amiresmaili (2018) | ^120^ | To assess the probable relationship between SES of Kerman city households and their children’s dental caries. | Yes | 25 | Cross-sectional (n=25) | Iran (city of Kerman) | Parents’ education  Parents’ occupation  Family income  Family SES | Prevalence of dental caries |
| Amjad (2019) | ^121^ | To evaluate the evidence on the association between social determinants of health and adverse maternal and birth outcomes in adolescent mothers. | Yes | Systematic review (n=31)  Meta-analysis (n=5) | Longitudinal (n=22)  Cross-sectional (n=7)  Case-control (n=1)  Ecological (n=1) | Adolescent mothers who gave birth before 20 years of age | Education  Occupation  SES (unclear definition) | Maternal outcomes (e.g., pre‐eclampsia, eclampsia, placenta previa, abruptio placentae, gestational diabetes mellitus, and intra‐hospital death)  Adverse birth outcome (e.g., low birth weight, perinatal death) |
| Bastos (2011) | ^122^ | To identify and to analyse research carried out in Brazil, investigating the association between periodontal outcomes and socioeconomic conditions. | No | 29 | Cross-sectional (n=28)  Case-control (n=1) | Brazil | Education (schooling)  Income  Combined SEP | Prevalence of gingivitis, periodontitis, and other periodontal conditions, and Community Periodontal Index |
| Best (2019) | ^123^ | To systematically review and appraise the existing evidence on the association between SES and mortality among children with congenital heart disease. | Yes | 28 | Not stated | Children with congenital heart disease | Parental and area-based education | Mortality |
| Bijker (2016) | ^124^ | To assess the influence of early-life conditions on cardiovascular disease, principally heart disease, and stroke and its risk factors in ethnic minority populations residing in Western countries. | No | 19 | Longitudinal (n=11)  Cross-sectional (n=8) | Ethnic minority populations residing in Western countries (i.e., the US, Canada, Western Europe, Australia and New Zealand) | Parents’ education  Parents’ occupation  Home ownership of parents  Number of rooms  Access to household assets  Height (marker of childhood SES) | Prevalence of cardiovascular diseases (heart failure and stroke) and risk factors (metabolic syndrome, obesity, hypertension and inflammatory risk markers) |
| Blumenshine (2010) | ^125^ | To examine the links between socioeconomic factors and birth outcomes, with specific attention to the strength and consistency of effects across socioeconomic measures, birth outcomes, and populations. | No | 106 | Longitudinal (n=20)  Cross-sectional (n=69)  Case-control (n=11)  Ecological (n=6) | OECD countries only | Education  Occupation  Income | Weight-related outcomes  Gestational age-related outcomes  Growth restriction outcomes |
| Boillot (2011) | ^126^ | To update systematic review of studies examining the association of educational attainment and chronic periodontitis in adults in the general population. | Yes | 18 | Longitudinal (n=2)  Cross-sectional (n=16) | General population  Adults aged 35+ | Education | Chronic periodontitis |
| Borschuk (2015) | ^127^ | To better understand health disparities in type 1 diabetes by race/ethnicity and SES. | No | 30 | Not stated | Children and adolescents with type 1 diabetes – age range 0-21 | Parents’ education  Household income  Composite score (Hollingshead index)  Insurance status | Health outcomes (hypoglycaemia, hyperglycaemia, and HbA1c levels)  Psychosocial outcomes (quality of life or symptoms of depression or anxiety) |
| Boss (2011) | ^128^ | To systematically review the scientific literature to assess for racial/ethnic or socioeconomic health disparities in the diagnosis and treatment of children with sleep-disordered breathing. | No | 33 | Longitudinal (n=14)  Cross-sectional (n=11)  Case-control (n=3)  Retrospective cohort (n=5) | Children (<18 years old) | Childhood SES  (unclear definition) | Prevalence of sleep-disordered breathing and decreased likelihood of undergoing adenotonsillectomy |
| Bougea (2022) | ^129^ | To explore the association between psychosocial stress (including socioeconomic inequalities) and late onset dementia. | Yes | Systematic review (n=24)  Meta-analysis (n=13) | Longitudinal (n=37) | Community dwelling population (at least 50% of the sample aged 65+)  Free of cognitive impairment and other psychiatric disorders at baseline | Education  Occupation  Income | Risk of dementia and Alzheimer’s disease |
| Boylan (2018) | ^130^ | To evaluate the evidence linking SES to blood pressure and heart rate responses to standardized stressors. | Yes | 26 | Not stated | Adolescents or adults (mean age at least 11 years) | Education  Occupation  Income  Composite score (Hollingshead index) | Cardiovascular responses to standardized stressors, including heart rate, systolic blood pressure, diastolic blood pressure. |
| Bridger Staatz (2021) | ^131^ | To assess the association between SEP and measures of body composition in the general population. | No | 47 | Not stated | General population  Adults | Parental education  Parental occupation  Parental income  Composite SES | Body composition |
| Bridger Staatz (2021) | ^132^ | To assess the association between SEP and measures of body composition in children from general population samples. | No | 50 | Not stated | General population  Children under 18 years of age | Parental education  Parental occupation  Parental income  Composite SES | Body composition |
| Burneo (2009) | ^133^ | To review the evidence on disparities in epilepsy with a focus on North American data. | No | 44  (Note: only 7 relevant to this study) | Longitudinal (n=2)  Cross-sectional (n=4)  Case-control (n=1) | Canada, the United States, and the English-speaking countries of the Caribbean region  Children and adults | Education  Occupation  Income | Epilepsy |
| Chung (2016) | ^134^ | To identify whether trends in overweight and obesity prevalence differ according to SEP among children and adolescents in economically advanced countries. | No | 30 | Not stated | Economically advanced countries (unclear definition)  General population of children and adolescents aged 2-18 years | Parents’ education  Parents’ occupation  Family income | Overweight or obesity |
| Conway (2008) | ^135^ | To quantitatively assess the association between SES and oral cancer incidence risk. | Yes | 41 | Case-control (n=41) | No restriction | Education  Occupation  Income  Household income | Oral cancer |
| Costa (2018) | ^136^ | To evaluate the effect modification of socioeconomic indicators on dental caries in adults. | Yes | Systematic review (n=61)  Meta-analysis (n=25) | Longitudinal (n=14)  Cross-sectional (n=45)  Case-control (n=1)  Ecological (n=1) | General population aged 19 to 60 years | Education (schooling)  Occupation  Income  Subjective SES  Composite score | Dental caries |
| Crichton (2015) | ^137^ | To examine variation in chlamydia prevalence in populations and possible sources of this variation. | Yes | Systematic review (n=36)  Meta-analysis (n=14) | Surveys with different sampling methods | Europe, North America, and Australia  Young adults aged 12-24 years | Education  Occupation  Parent’s education  Parent’s occupation  Parent’s income | Chlamydia infection |
| Cundiff (2017) | ^138^ | To evaluate whether subjective SES offers additional utility in accounting for variance in physical health measures above and beyond well-established objective measures of SES in adults. | Yes | 31 | Longitudinal (n=4)  Cross-sectional (n=27) | Adults aged 18 years and over | Subjective SEP | Self-rated health  Quality of life  Physiologic measures (e.g., blood pressure, cortisol)  Disease diagnoses (e.g., hypertension, diabetes)  Physical symptoms (e.g., fatigue, headache). |
| Degarege (2019) | ^139^ | To summarise literature on the effect of house structure, education level, occupation, income, and wealth on the epidemiology of malaria in all age groups in sub-Saharan Africa. | Yes | Systematic review (n=84)  Meta-analysis (n=75) | Longitudinal (n=12)  Cross-sectional (n=57)  Case-control (n=10)  RCT (n=5) | Sub-Saharan Africa  All age groups | Education  Occupation  Income  Wealth  House structure | Prevalence and incidence of malaria infection |
| Didsbury (2016) | ^140^ | To synthesise the available evidence regarding the association between socioeconomic indicators and health‐related quality of life of children. | No | 30 | Longitudinal (n=1)  Cross-sectional (n=29) | Children and young adults (aged 2-21 years) having epilepsy, chronic kidney disease, type 1 diabetes, or asthma | Parents’ education  Parents’ occupation  Household income | Quality of life |
| El-Sayed (2012) | ^141^ | To review the literature about socioeconomic inequalities in childhood obesity in the United Kingdom. | No | 23 | Longitudinal (n=5)  Cross-sectional (n=18) | United Kingdom  Children aged <18 years | Parents’ education  Parents’ occupation  Household income | Childhood obesity |
| El-Sayed (2012) | ^142^ | To summarize important differences in the prevalence and determinants of obesity by different indicators of SEP in the United Kingdom. | No | 35 | Longitudinal (n=20)  Cross-sectional (n=15) | United Kingdom  Adults  (>18years old) | Education  Occupation  Income  Parental SEP | Adult obesity |
| Elwadhi (2020) | ^143^ | To identify, review, and synthesize evidence on whether social disadvantage moderates antidepressant treatment outcomes, even when access to treatment is not a consideration. | No | 9 studies  (13 publications) | Uncontrolled trials (n=4)  RCT (n=2)  Cluster RCT (n=1)  Trials with sequential non- and randomized phases (n=2) | Adults  (>18years old) who were receiving treatment for diagnosed major depression | Education  Employment status  Income  Household income | Antidepressant treatment outcomes (e.g., response, remission, or recovery) |
| Etindele Sosso (2021) | ^144^ | To systematically summarize the evidence from the existing literature on the association between SES and obstructive sleep apnea. | No | 11 | Longitudinal (n=1)  Cross-sectional (n=10) | General population | Education  Occupation  Income  Social class  Maternal education Maternal employment  Household income | Obstructive sleep apnea |
| Fruhstorfer (2016) | ^145^ | To examine the association between overweight and obesity among school‐age children in sub‐Saharan Africa with SES. | Yes | 20 | Cross-sectional (n=20) | Sub-Saharan Africa  Children and adolescents aged 5-18 years | Parental education  Parental occupation  Parental income  Parental assets | Prevalence of overweight and obesity |
| Fryers (2003) | ^146^ | To provide a systematic review of published evidence on the links between the range of conventional markers of social position and the common mental disorders in the general population in developed countries. | No | 9 | Not stated | General population from developed countries  Adults of working age | Education  Occupation  Employment status  Income | Mental health disorders |
| Galobardes (2006) | ^147^ | To present a systematic review of the evidence on whether adverse socioeconomic circumstances in childhood confer greater risk for cardiovascular disease and its specific subcategories. | No | 40 studies (50 publications) | Longitudinal (n=24)  Cross-sectional (n=5)  Case-control (n=11) | No restriction | Parental education  Father’s occupation  Parents’ unemployment  Housing condition | Morbidity and mortality from cardiovascular disease and specific disease subtypes, including coronary heart disease, ischemic and haemorrhagic stroke, peripheral vascular disease, markers of atherosclerosis, and rheumatic heart disease |
| Gershon (2012) | ^148^ | To examine the consistency and magnitude of the association between socioeconomic status and Chronic Obstructive Pulmonary Disease. | No | 15 | Not stated | Adults either diagnosed with or at risk of Chronic Obstructive Pulmonary Disease. | Education  Occupation  Income | Chronic Obstructive Pulmonary Disease prevalence, incidence, prognosis, and health care utilization |
| Goulden (2015) | ^149^ | To determine whether SES is related to the risk of developing Multiple sclerosis. | No | 21 | Longitudinal (n=2)  Cross-sectional (n=19) | No restrictions | Education  Occupation  Income  Wealth  Parents’ education  Parents’ occupation  Home ownership of parents | Multiple sclerosis |
| Grintsova (2014) | ^150^ | To synthesize the current evidence, focusing on process and intermediate outcome indicators of health care for patients with Type 2 diabetes and on differences by individual SES and residential area deprivation. | No | 21 | Longitudinal (n=9)  Cross-sectional (n=12) | Population comprises type 2 diabetes patients or a mixed group of type 1 and type 2 diabetes patients | Education  Occupation  Income | Intermediate outcome indicators HbA1c, blood pressure, and body mass index |
| Guglielmi (2019) | ^151^ | To systematically analyse the literature for an association between obstructive sleep apnea, SES and race/ethnicity in adults. | No | 17 | Longitudinal (n=1)  Cross-sectional (n=16) | Adults | Education  Occupation  Income  Household income | Obstructive sleep apnea |
| Hanna (2017) | ^152^ | To report the health inequalities facing stroke survivors in the United Kingdom and Ireland with visual impairments as described in the current literature. | No | 65 | Not stated | United Kingdom and Ireland  Adult participants (aged 18 years or over) diagnosed with a stroke or a visual impairment | Unclear; included:  Education  Occupation  Income | Unclear, included:  Clinical improvement in visual functions, functional improvement in activities of daily living, quality-of-life measures, occurrence of stroke and visual impairment, mortality. |
| Houweling (2016) | ^153^ | To bring together the recent evidence on the distribution of Neglected Tropical Diseases prevalence—and/or prevalence of the underlying infection—across socioeconomic strata. | No | 93 | Not stated | Top-20 country in terms of infection burden for the Neglected Tropical Diseases under study | Unclear; included:  Educational  Occupation  Economic status  Household ownership of assets  Socioeconomic hierarchy | Nine NTDs listed in the London Declaration for intensified control and/or elimination, including those that are controlled through preventive chemotherapy (i.e., lymphatic filariasis, onchocerciasis, schistosomiasis, soil-transmitted helminths, and trachoma) and those controlled through intensified disease management (i.e., Chagas' disease, human African trypanosomiasis, leprosy, and visceral leishmaniasis). |
| Karanth (2019) | ^154^ | To synthesize the current scientific evidence regarding the role of race, SES, and access to care on ovarian cancer treatment and survival. | Yes | 41 | Not stated | United States  Female | Education  Income  Poverty | Ovarian cancer treatment and mortality |
| Kim (2013) | ^155^ | To understand variation in infant mortality and birth outcomes across and within the United States of America and Western Europe, by conceptualizing a social determinants of infant mortality/birth outcomes framework. | No | Not stated | Not stated | United States and Western Europe | Individual and parental SES (unclear definition) | Infant mortality and birth outcomes (fetal mortality, neonatal mortality, preterm birth, low birth weight) |
| Knorst (2021) | ^156^ | To systematically review observational studies assessing the association between socioeconomic status and oral health-related quality of life in children, adolescents and adults. | Yes | Systematic review (n=139)  Meta-analysis (n=75) | Longitudinal (n=12)  Cross-sectional (n=127) | General population of all ages.  Studies that included particularly vulnerable  subpopulations. | Education  Occupation  Household income  Socioeconomic indices (e.g., social deprivation index) | Oral health-related quality of life |
| Kolahdooz (2015) | ^157^ | To examine the current SES and material circumstances facing Indigenous Canadians in the province of Alberta. | No | 25 | Not stated | Indigenous Canadians in Alberta, Canada | Education  Employment status  Housing | Health outcome not specified |
| Lago (2018) | ^158^ | To investigate the role of socioeconomic variables, their composition and distribution according to health status, particularly on non-communicable diseases. | No | 26 reported only | Not stated | OECD countries | SES (unclear definition; mainly considered Income) | Non-communicable diseases  Health status |
| Lee (2021) | ^159^ | To examine the nature of socioeconomic disparities in cardiovascular health in South Korea. | No | 42 | Not stated | South Korea  Adults older than 19 years old | Education  Occupation  Employment status  Income  Composite SES | Cardiovascular conditions  Sub-risk factors of cardiovascular diseases  Cardiovascular mortality |
| Li (2021) | ^160^ | To evaluate the evidence on the association of SES and melanoma incidence in Canada. | No | 7 | Longitudinal (n=5)  Case-control (n=2) | Canada | Income  Occupation  Marginalization index | Melanoma incidence |
| Lindner (2018) | ^161^ | To examine the associations of individual-level as well as area-level SES and area-level deprivation with glycaemic control, hypoglycaemia and diabetic ketoacidosis in people with type 1 diabetes. | No | 22 | Longitudinal (n=10)  Cross-sectional (n=12) | Australia, Canada, Europe, New Zealand and the US  People with type 2 diabetes | Education  Occupation  Income  Parental SES | Glycaemic control  Hypoglycaemia  Diabetic ketoacidosis |
| Liu (2017) | ^162^ | To systematically review the association between early life SES and subsequent adult markers of inflammation, with a focus on C-reactive protein. | Yes | 21 | Longitudinal (n=16)  Cross-sectional (n=5) | Children and adults | Parental education  Parental occupation  Parental income | Inflammatory markers (e.g., C-reactive protein, interleukin  and fibrinogen) |
| Lorant (2003) | ^163^ | To measure the magnitude and shape of the association between SES and depression. | Yes | 56 | Not stated | Population-based community sample  Adults aged 16 years and over | Education  Occupation  Income  Social class  Wealth | Prevalence, incidence, and persistence of major depression |
| Lund (2019) | ^164^ | To investigate whether socioeconomic status is associated with stress and/or depressive symptoms in the general population of adolescent girls. | No | 8 | Cross-sectional (n=8) | Western countries (Europe, North America, Australia and New Zealand)  Adolescent girls aged 13-18 years in general population | Parental education  Parental unemployment  Family affluence  Perceived financial difficulties | Stress and depressive symptoms |
| Lundqvist (2016) | ^165^ | To summarise the published literature on the association between SES and breast cancer incidence, case fatality, and mortality in European women. | Yes | 25 | Longitudinal (n=23)  Cross-sectional (n=2) | European OECD countries  Female | Education  Occupation  Income | Female breast cancer incidence  Case fatality Mortality |
| Lyle (2017) | ^166^ | To gather a weight of evidence to determine the strength and direction of the SES relationships across the breast cancer continuum. | No | 44 | Not stated | Australia  Female | Education  Income | Incidence, detection,  diagnosis, treatment, survival and mortality along the breast cancer continuum. |
| Mahboob (2021) | ^167^ | To examine the association between SES and unintentional injuries among children and adolescents. | No | 54 | Longitudinal (n=19)  Cross-sectional (n=20)  Case-control (n=9)  Ecological (n=6) | Children and adolescents (0-19 years old) | Parental education  Parental income  Household size | Measures of unintentional injury, including mortality, hospitalizations, injury reports, and Emergency Department visits. |
| Manrique-Garcia (2011) | ^168^ | To investigate the association between acute  myocardial infarction incidence and various SEP measures. | Yes | 70 studies (65 publications) | Longitudinal (n=30)  Case-control (n=35) | No restrictions | Education  Occupation  Income | Acute myocardial infarction incidence |
| Mihor (2020) | ^169^ | To review studies that have assessed the direction and magnitude of socioeconomic inequalities in location-specific cancer incidence among European adults. | No | 91 | Not stated (mostly longitudinal) | Europe  Adults | Education  Occupation  Income  Social class  Housing and car ownership | Cancer incidence |
| Morton (2016) | ^170^ | To systematically review the literature for evidence of a social gradient in health for adults with moderate-to-severe chronic kidney disease. | No | 58 | Longitudinal (n=51)  Cross-sectional (n=5)  RCT (n=2) | Adults | Education  Occupation  SES (unclear definition)  Home ownership | Access to healthcare  Kidney disease progression Cardiovascular events  All-cause mortality |
| Muscatell (2020) | ^171^ | To quantify the association between SES and two markers of systemic inflammation, C-reactive protein, interleukin-6. | Yes | 43 | Not stated | General North American population without an existing chronic condition or disease | Education  Occupation  Income  Subjective SES | C-reactive protein  Interleukin-6 |
| O’Brien (2018) | ^172^ | To summarize current evidence relating to the association between SES and gestational weight gain. | No | 16 | Longitudinal (n=9)  Cross-sectional (n=5)  Retrospective (n=2) | Pregnant women aged >18 years with a singleton pregnancy | Education  Occupation  Income  Social class  Deprivation  Poverty | Gestational weight gain |
| Ombrellaro (2018) | ^173^ | To consolidate evidence regarding the association between SES and cardiorespiratory fitness. | Yes | Systematic review (n=15)  Meta-analysis (n=3) | Longitudinal (n=1)  Cross-sectional (n=14) | Adults | Education  Occupation  Income  Employment status  Composite SES | Cardiorespiratory fitness |
| Pan (2020) | ^174^ | To evaluate the effects of SES on hyperuricemia and gout quantitatively. | Yes | 14 | Longitudinal (n=5)  Cross-sectional (n=9) | General population | Education  Occupation  Income  Household wealth | Prevalence of hyperuricemia or gout |
| Parikh (2003) | ^175^ | To investigate the relationship between cervical cancer and socioeconomic characteristics separately for stage of disease, geographical region, age and histological type. | Yes | 76 | Case-control (n=76) | No restrictions | Education  Income  Social class  Composite SES | Cervical cancer or dysplasia |
| Petridou (2015) | ^176^ | To evaluate whether SES is associated with survival from childhood leukemia. | Yes | 29 | Longitudinal (n=29) | Children | Education  Family income  Maternal education  Parental occupation | Leukemia  Overall survival |
| Peykari (2015) | ^177^ | To describe the cross-sectional association between socioeconomic factors and diabetes in Iranian population. | No | 15 | Cross-sectional (n=15) | General population from Iran | Education  Income  Occupation | Diabetes prevalence, control, and complications |
| Pillas (2014) | ^178^ | To identify the key social factors operating at the household, neighbourhood, and country levels that drive inequalities in child health and development. | No | 201 | Not stated | WHO European Region (53 countries)  Prenatal development to 8 years of age | Parental education  Parental occupation  Parental income  Household level SES | Wide range of childhood developmental outcomes (e.g.,  mortality, general health/illness, paediatric cancers, allergies, respiratory diseases, mental health, pre-and perinatal, neurological disorders, infections, anthropometric, digestive disease) |
| Probst (2014) | ^179^ | To examine socioeconomic differences in alcohol-attributable and all-cause mortality. | Yes | 15 | Not stated | General population  Aged 15+ years old | Education  Occupation  Income  Employment status | Alcohol-attributable mortality  All-cause mortality |
| Quon (2014) | ^180^ | To examine the association between subjective SES and health outcomes during adolescence. | Yes | 44 | Not stated | Adolescents age range 12-19 years | Subjective SES | Self-rated health  Mental health  Physical health  Health behaviours |
| Read (2016) | ^181^ | To ascertain to what extent there is evidence of similar inequalities in the subjective health and well-being of older people in Europe. | No | 71 | Longitudinal (n=6)  Cross-sectional (n=65) | General population from Europe  Older adults aged 60+ | Education  Occupation  Income  Wealth  Home or car ownership  Social class  Housing tenure | Self-rated health  Quality of life  Life satisfaction |
| Schwendicke (2015) | ^182^ | To evaluate the possible association between SEP and caries. | Yes | Systematic review (n=155)  Meta-analysis (n=92) | Longitudinal (n=24)  Cross-sectional (n=122)  Case-control (n=9) | Adults or children with permanent or deciduous teeth | Education  Occupation  Income  Parental education  Parental occupation  Parental income | Caries severity  Caries incidence |
| Scott (2017) | ^183^ | To carry out a systematic review of socioeconomic inequalities in mortality, morbidity, and diabetes management solely in relation to adults with type 1 diabetes. | No | 33 | Longitudinal (n=12)  Cross-sectional (n=19)  Case-control (n=2) | OECD countries  Adults aged 16 years and over with type 1 diabetes | Education  Income  Social status | Mortality  Morbidity arising from diabetes  Diabetes management |
| Senese (2009) | ^184^ | To summarize associations between childhood SEP and adulthood obesity. | No | 48 | Longitudinal (n=8)  Cross-sectional (n=40) | No restrictions | Parental education  Parental occupation | Adult obesity |
| Sidorchuk (2009) | ^185^ | To investigate the social inequality in lung cancer incidence. | Yes | 64 studies (63 publications) | Not stated | No restrictions | Education  Occupation  Income | Lung cancer incidence |
| Silva (2014) | ^186^ | To perform a systematic review of articles published on the social determinants associated with mortality in the elderly. | No | 20 | Longitudinal (n=20) | Older adults aged 60 years and over | Education  Unemployment | Mortality |
| Spencer (2015) | ^187^ | To determine the association of socioeconomic disadvantage with the prevalence of childhood disabling chronic conditions in high-income countries. | Yes | Systematic review (n=160)  Meta-analysis (n=126) | Longitudinal (n=25)  Cross-sectional (n=90)  Case-control (n=16)  Routine data (n=21)  Disease registries (n=8) | General population from high-income OECD countries  Mainly children age range 0-18 years; 11 studies included young people aged 19-21 years | Parental education  Parental occupation  Parental income  Housing tenure  Composite SES | All-cause disabling chronic conditions  Psychological disorders  Intellectual disability  Sensory impairments  Congenital abnormalities  Specific conditions, such as asthma, cerebral palsy and epilepsy |
| Tamayo (2010) | ^188^ | To evaluate the risk of psychosocial factors on type 2 diabetes incidence and the role of change in socioeconomic conditions throughout life. | No | Type 2 diabetes (n=10) Obesity (n= 14) | Longitudinal (n=24) | General population | Parental education  Parental occupation  Family income | Type 2 diabetes incidence  Development of metabolic  disorders and obesity |
| Tang (2016) | ^189^ | To determine the association between subjective social status and the odds of coronary artery disease, hypertension, diabetes, obesity, and dyslipidaemia. | Yes | 10 | Longitudinal (n=1)  Cross-sectional (n=9) | Adults aged 18 years and over | Subjective social status | Coronary artery disease Hypertension  Diabetes  Obesity  Dyslipidaemia |
| Tatulashvili (2020) | ^190^ | To evaluate the risk of micro- and macrovascular complications in patients with type 2 diabetes according to SES in high-income countries. | No | 28 | Longitudinal (n=9)  Cross-sectional (n=19) | High-income countries  Adult patients (19 years old or older) with type 2 diabetes | Education  Income  Composite SES | Diabetes complications (e.g., retinopathy, cardiovascular complications, stroke, nephropathy, neuropathy, amputation) |
| Thomson (2021) | ^191^ | To understand the association between individual-level and household-level measures of SES and adverse pregnancy outcomes. | Yes | 35 | Longitudinal (n=25)  Case-control (n=10) | The unborn fetus, neonate (child under 28 days of age) or mother in the United Kingdom or Ireland | Education  Occupation  Income  Employment status  Financial status/hardship  Household-level SES | Pregnancy outcomes related to the fetus, neonate or mother |
| Uphoff (2015) | ^192^ | To clarify associations between SEP and the prevalence of asthma and allergies. | Yes | 183 | Longitudinal (n=47)  Cross-sectional (n=124)  Case-control (n=8)  Time series (n=4) | No restrictions | Education  Occupation  Income  Wealth  Employment status  Parental education  Parental income | Prevalence of asthma and allergies |
| Uthman (2013) | ^193^ | To identify studies that examined gastric cancer incidence in relation to SEP. | Yes | 36 | Longitudinal (n=13)  Case-control (n=23) | Patients with diagnosed gastric cancer | Education  Occupation  Income  Composite SEP | Incidence of gastric cancer |
| Valentin (2020) | ^194^ | To investigate the association between measures of SES and post- fracture outcomes across the world. | Yes | Overall (n=24)  Mortality (n=20) Health-related quality of life (n=4) | Longitudinal (n=24) | No restrictions | Education  Occupation  Income | Mortality  Health-related quality of life following a fragility fracture |
| Vart (2015) | ^195^ | To summarize the association between SES and chronic kidney disease. | Yes | 35 | Longitudinal (n=10)  Cross-sectional (n=21)  Case-control (n=3) | General population older than 18 | Education  Occupation  Individual/household/family Income  Wealth/assets  Poverty level  Composite SES  Area-level SES | Chronic kidney disease |
| Wagg (2021) | ^196^ | To assess the association between SEP and healthy ageing. | No | 45 | Longitudinal (n=19)  Cross-sectional (n=26) | Middle-aged and  community-dwelling older adults | Education  Occupation  Income  Personal or household wealth  Housing condition  Composite SEP | Healthy ageing (a composite measure of two or more healthy ageing domains) |
| Weightman (2012) | ^197^ | To determine the association between area and individual measures of social disadvantage and infant health in the UK. | Yes | 36 | Not stated | General population  Infants (0-12 months) in the UK | Parental education  Parental occupation  Household income/poverty | Preterm birthBirth weight  Mortality  Diagnosed illness  Attendance at primary or secondary care in relation to ill health  Infection  Injury or disability  Growth and development |
| Williams (2018) | ^198^ | To map the literature on evidence from low- and lower-middle-income countries on the SES gradient of four particular non-communicable diseases: cardiovascular disease, cancer, diabetes, and chronic respiratory diseases. | No | 57 | Longitudinal (n=18)  Cross-sectional (n=30)  Case-control (n=9) | General population  Low- and lower middle-income countries as defined by World Bank | Education  Occupation  Income  Social class  Access to basic needs  Household wealth  Composite SES | Morbidity or mortality from cardiovascular disease, cancer, diabetes, and chronic respiratory diseases |
| Wojcicki (2005) | ^199^ | To examine the relationship between SES and HIV infection in women in sub-Saharan Africa at the individual, household and ecological level. | No | 36 | Longitudinal (n=5)  Cross-sectional (n=28)  Case-control (n=1)  Ecological (n=2) | East, Central and Southern Africa  Female | Education  Occupation  Employment status  Household income  Husband/parental education  Assets | HIV infection |
| Wu (2017) | ^200^ | To describe the association between SES and prevalence of type 2 diabetes in Chinese populations in mainland China, Hong Kong, and Taiwan. | No | 33 | Cross-sectional (n=33) | Chinese populations in mainland China, Hong Kong or Taiwan  Adults | Education  Occupation  Income | Type 2 diabetes |
| van Zwieten (2018) | ^201^ | To evaluate the effect of age on the association between household income and overall health status from birth to adolescence. | No | 43 | Longitudinal (n=13)  Cross-sectional (n=30) | Children and adolescents (0-18 years of age) | Parental/family/household income  Income poverty | Single-item subjective overall health status |

*Abbreviations*: OECD, Organisation for Economic Co-operation and Development; RCT, randomized controlled trial; SES, socioeconomic status; SEP, socioeconomic position; UK, United Kingdom; US, United States.

## Table S3. Critical appraisal for included studies using AMSTAR 2

| First Author (Year) | Citation | 1 | 2 | 3 | 4 | 5 | 6 | 7 | 8 | 9  RCT^#^ | 9  NRS | 10 | 11  RCT^#*^ | 11  NRS^*^ | 12^*^ | 13 | 14 | 15^*^ | 16 |
| --- | --- | --- | --- | --- | --- | --- | --- | --- | --- | --- | --- | --- | --- | --- | --- | --- | --- | --- | --- |
| Adams (2018) | ^118^ | 0 | 1 | 0 | 0 | 2 | 2 | 2 | 0 | NR | 0 | 0 | NR | 0 | 2 | 2 | 2 | 2 | 2 |
| Adegbosin (2019) | ^119^ | 2 | 1 | 0 | 0 | 2 | 0 | 0 | 0 | 0 | 1 | 0 | 0 | 0 | 2 | 0 | 0 | 2 | 2 |
| Amiresmaili (2018) | ^120^ | 2 | 0 | 0 | 1 | 2 | 0 | 0 | 1 | NR | 1 | 0 | NR | 0 | 0 | 0 | 0 | 2 | 2 |
| Amjad (2019) | ^121^ | 0 | 0 | 0 | 1 | 2 | 2 | 0 | 0 | NR | 1 | 0 | NR | 0 | 0 | 2 | 2 | 0 | 0 |
| Bastos (2011) | ^122^ | 2 | 0 | 2 | 0 | 2 | 2 | 0 | 1 | NR | 0 | 0 | NR | NR | NR | 0 | 0 | NR | 0 |
| Best (2019) | ^123^ | 2 | 0 | 0 | 0 | 2 | 2 | 0 | 0 | NR | 1 | 0 | NR | 2 | 0 | 2 | 0 | 0 | 0 |
| Bijker (2016) | ^124^ | 0 | 0 | 2 | 0 | 0 | 0 | 0 | 1 | NR | 0 | 0 | NR | NR | NR | 0 | 2 | NR | 2 |
| Blumenshine (2010) | ^125^ | 0 | 0 | 2 | 0 | 2 | 0 | 0 | 1 | NR | 0 | 0 | NR | NR | NR | 2 | 2 | NR | 2 |
| Boillot (2011) | ^126^ | 2 | 0 | 2 | 0 | 2 | 2 | 0 | 1 | NR | 0 | 0 | NR | 2 | 0 | 0 | 2 | 2 | 2 |
| Borschuk (2015) | ^127^ | 2 | 0 | 0 | 0 | 0 | 0 | 0 | 1 | NR | 0 | 0 | NR | NR | NR | 0 | 0 | NR | 0 |
| Boss (2011) | ^128^ | 0 | 0 | 0 | 0 | 2 | 0 | 0 | 0 | NR | 0 | 0 | NR | NR | NR | 0 | 0 | NR | 2 |
| Bougea (2022) | ^129^ | 2 | 0 | 2 | 0 | 2 | 2 | 0 | 2 | NR | 1 | 0 | NR | 2 | 0 | 2 | 2 | 2 | 2 |
| Boylan (2018) | ^130^ | 2 | 0 | 0 | 0 | 0 | 2 | 1 | 0 | NR | 0 | 0 | NR | 0 | 0 | 0 | 2 | 2 | 2 |
| Bridger Staatz (2021) | ^131^ | 2 | 1 | 0 | 0 | 2 | 2 | 0 | 0 | NR | 1 | 0 | NR | NR | NR | 0 | 2 | NR | 2 |
| Bridger Staatz (2021) | ^132^ | 2 | 1 | 0 | 0 | 2 | 2 | 0 | 0 | NR | 1 | 0 | NR | NR | NR | 2 | 2 | NR | 2 |
| Burneo (2009) | ^133^ | 0 | 0 | 0 | 1 | 2 | 0 | 0 | 0 | NR | 0 | 0 | NR | NR | NR | 0 | 0 | NR | 2 |
| Chung (2016) | ^134^ | 2 | 1 | 0 | 0 | 2 | 2 | 0 | 0 | NR | 0 | 0 | NR | NR | NR | 2 | 2 | NR | 2 |
| Conway (2008) | ^135^ | 2 | 0 | 2 | 0 | 2 | 0 | 0 | 1 | NR | 1 | 0 | NR | 0 | 2 | 2 | 2 | 2 | 0 |
| Costa (2018) | ^136^ | 2 | 0 | 0 | 1 | 2 | 2 | 0 | 1 | NR | 1 | 0 | NR | 0 | 0 | 0 | 0 | 0 | 2 |
| Crichton (2015) | ^137^ | 2 | 0 | 0 | 1 | 2 | 0 | 0 | 0 | NR | 1 | 2 | NR | 0 | 2 | 2 | 2 | 0 | 2 |
| Cundiff (2017) | ^138^ | 2 | 0 | 0 | 0 | 0 | 2 | 0 | 1 | NR | 0 | 0 | NR | 0 | 0 | 0 | 2 | 2 | 0 |
| Degarege (2019) | ^139^ | 2 | 0 | 0 | 1 | 2 | 2 | 0 | 0 | 1 | 1 | 0 | 0 | 0 | 2 | 2 | 2 | 2 | 2 |
| Didsbury (2016) | ^140^ | 2 | 0 | 2 | 1 | 0 | 2 | 0 | 1 | NR | 0 | 0 | NR | NR | NR | 2 | 2 | NR | 2 |
| El-Sayed (2012) | ^141^ | 2 | 0 | 2 | 0 | 0 | 0 | 0 | 1 | NR | 0 | 0 | NR | NR | NR | 0 | 0 | NR | 2 |
| El-Sayed (2012) | ^142^ | 2 | 0 | 2 | 0 | 0 | 0 | 0 | 1 | NR | 0 | 0 | NR | NR | NR | 0 | 0 | NR | 2 |
| Elwadhi (2020) | ^143^ | 0 | 0 | 2 | 0 | 0 | 0 | 0 | 2 | 1 | 1 | 0 | NR | NR | NR | 2 | 0 | NR | 2 |
| Etindele Sosso (2021) | ^144^ | 2 | 0 | 2 | 0 | 0 | 0 | 0 | 1 | NR | 0 | 0 | NR | NR | NR | 0 | 0 | NR | 2 |
| Fruhstorfer (2016) | ^145^ | 0 | 0 | 0 | 1 | 2 | 2 | 0 | 0 | NR | 0 | 0 | NR | 0 | 0 | 2 | 2 | 2 | 0 |
| Fryers (2003) | ^146^ | 0 | 0 | 0 | 0 | 0 | 0 | 0 | 0 | NR | 0 | 0 | NR | NR | NR | 0 | 2 | NR | 2 |
| Galobardes (2006) | ^147^ | 0 | 0 | 0 | 0 | 0 | 0 | 0 | 0 | NR | 0 | 0 | NR | NR | NR | 0 | 0 | NR | 0 |
| Gershon (2012) | ^148^ | 2 | 0 | 0 | 0 | 2 | 2 | 0 | 0 | NR | 1 | 0 | NR | NR | NR | 2 | 2 | NR | 2 |
| Goulden (2015) | ^149^ | 0 | 0 | 2 | 1 | 2 | 0 | 0 | 0 | NR | 1 | 0 | NR | NR | NR | 2 | 2 | NR | 2 |
| Grintsova (2014) | ^150^ | 2 | 0 | 0 | 0 | 2 | 2 | 0 | 0 | NR | 1 | 0 | NR | NR | NR | 2 | 2 | NR | 2 |
| Guglielmi (2019) | ^151^ | 0 | 0 | 0 | 0 | 2 | 0 | 0 | 0 | NR | 0 | 0 | NR | NR | NR | 0 | 0 | NR | 2 |
| Hanna (2017) | ^152^ | 0 | 0 | 0 | 1 | 2 | 2 | 0 | 0 | 0 | 0 | 0 | NR | NR | NR | 0 | 0 | NR | 2 |
| Houweling (2016) | ^153^ | 0 | 0 | 0 | 1 | 0 | 2 | 0 | 2 | NR | 0 | 0 | NR | NR | NR | 0 | 2 | NR | 2 |
| Karanth (2019) | ^154^ | 2 | 0 | 0 | 1 | 2 | 0 | 0 | 0 | NR | 0 | 0 | NR | 0 | 0 | 0 | 2 | 0 | 2 |
| Kim (2013) | ^155^ | 0 | 0 | 0 | 0 | 0 | 0 | 0 | 0 | 0 | 0 | 0 | NR | NR | NR | 2 | 0 | NR | 2 |
| Knorst (2021) | ^156^ | 2 | 0 | 0 | 1 | 2 | 2 | 0 | 1 | NR | 1 | 0 | NR | 0 | 2 | 2 | 2 | 2 | 2 |
| Kolahdooz (2015) | ^157^ | 0 | 0 | 0 | 0 | 2 | 0 | 0 | 0 | 0 | 0 | 0 | NR | NR | NR | 0 | 0 | NR | 2 |
| Lago (2018) | ^158^ | 0 | 0 | 0 | 1 | 0 | 0 | 0 | 0 | NR | 0 | 0 | NR | NR | NR | 0 | 0 | NR | 2 |
| Lee (2021) | ^159^ | 2 | 0 | 0 | 0 | 2 | 0 | 0 | 1 | NR | 0 | 0 | NR | NR | NR | 0 | 2 | NR | 2 |
| Li (2021) | ^160^ | 2 | 1 | 2 | 0 | 2 | 2 | 0 | 0 | NR | 1 | 0 | NR | NR | NR | 2 | 2 | NR | 2 |
| Lindner (2018) | ^161^ | 2 | 1 | 2 | 0 | 0 | 0 | 0 | 1 | NR | 0 | 0 | NR | NR | NR | 0 | 2 | NR | 2 |
| Liu (2017) | ^162^ | 2 | 1 | 2 | 1 | 2 | 2 | 0 | 1 | NR | 0 | 0 | NR | 2 | 0 | 2 | 2 | 2 | 2 |
| Lorant (2003) | ^163^ | 2 | 0 | 0 | 1 | 0 | 0 | 2 | 0 | NR | 0 | 0 | NR | 0 | 2 | 2 | 2 | 2 | 0 |
| Lund (2019) | ^164^ | 2 | 0 | 0 | 2 | 0 | 0 | 0 | 0 | NR | 0 | 0 | NR | NR | NR | 0 | 2 | NR | 0 |
| Lundqvist (2016) | ^165^ | 2 | 0 | 0 | 0 | 2 | 2 | 0 | 1 | NR | 1 | 0 | NR | 0 | 0 | 2 | 0 | 2 | 2 |
| Lyle (2017) | ^166^ | 2 | 0 | 2 | 1 | 0 | 0 | 0 | 0 | NR | 0 | 0 | NR | NR | NR | 2 | 0 | NR | 2 |
| Mahboob (2021) | ^167^ | 0 | 0 | 0 | 0 | 0 | 0 | 0 | 0 | NR | 1 | 0 | NR | NR | NR | 0 | 2 | NR | 0 |
| Manrique-Garcia (2011) | ^168^ | 2 | 0 | 0 | 0 | 2 | 0 | 0 | 0 | NR | 0 | 0 | NR | 0 | 0 | 0 | 2 | 2 | 2 |
| Mihor (2020) | ^169^ | 2 | 0 | 2 | 1 | 2 | 0 | 0 | 1 | NR | 0 | 0 | NR | NR | NR | 0 | 0 | NR | 2 |
| Morton (2016) | ^170^ | 2 | 0 | 0 | 1 | 0 | 0 | 0 | 1 | 0 | 1 | 0 | NR | NR | NR | 2 | 0 | NR | 2 |
| Muscatell (2020) | ^171^ | 2 | 0 | 0 | 0 | 0 | 2 | 0 | 2 | NR | 0 | 0 | NR | 0 | 0 | 0 | 2 | 2 | 0 |
| O’Brien (2018) | ^172^ | 2 | 1 | 2 | 0 | 2 | 2 | 0 | 0 | NR | 1 | 0 | NR | NR | NR | 2 | 0 | NR | 2 |
| Ombrellaro (2018) | ^173^ | 2 | 1 | 2 | 1 | 2 | 2 | 0 | 0 | NR | 1 | 2 | NR | 2 | 2 | 2 | 2 | 0 | 2 |
| Pan (2020) | ^174^ | 2 | 0 | 0 | 1 | 2 | 2 | 0 | 0 | NR | 1 | 0 | NR | 0 | 0 | 2 | 2 | 0 | 2 |
| Parikh (2003) | ^175^ | 0 | 0 | 0 | 0 | 0 | 0 | 0 | 0 | NR | 0 | 0 | NR | 0 | 0 | 0 | 2 | 0 | 0 |
| Petridou (2015) | ^176^ | 2 | 1 | 2 | 0 | 2 | 2 | 0 | 2 | NR | 0 | 0 | NR | 2 | 0 | 0 | 2 | 2 | 2 |
| Peykari (2015) | ^177^ | 2 | 0 | 2 | 1 | 0 | 0 | 0 | 0 | NR | 0 | 0 | NR | NR | NR | 0 | 0 | NR | 2 |
| Pillas (2014) | ^178^ | 0 | 0 | 0 | 0 | 2 | 0 | 0 | 0 | NR | 0 | 0 | NR | NR | NR | 0 | 0 | NR | 0 |
| Probst (2014) | ^179^ | 2 | 1 | 0 | 0 | 0 | 0 | 0 | 0 | NR | 0 | 0 | NR | 0 | 0 | 2 | 2 | 2 | 2 |
| Quon (2014) | ^180^ | 2 | 0 | 0 | 0 | 0 | 2 | 0 | 0 | NR | 2 | 0 | NR | 0 | 2 | 0 | 2 | 2 | 0 |
| Read (2016) | ^181^ | 0 | 0 | 2 | 1 | 2 | 2 | 0 | 0 | NR | 0 | 0 | NR | NR | NR | 0 | 0 | NR | 2 |
| Schwendicke (2015) | ^182^ | 2 | 1 | 2 | 0 | 2 | 0 | 2 | 0 | NR | 0 | 0 | NR | 0 | 2 | 2 | 2 | 2 | 2 |
| Scott (2017) | ^183^ | 2 | 0 | 2 | 0 | 2 | 0 | 0 | 1 | NR | 1 | 0 | NR | NR | NR | 2 | 2 | NR | 2 |
| Senese (2009) | ^184^ | 2 | 0 | 2 | 1 | 2 | 0 | 0 | 1 | NR | 0 | 0 | NR | NR | NR | 2 | 2 | NR | 2 |
| Sidorchuk (2009) | ^185^ | 2 | 0 | 0 | 0 | 2 | 0 | 0 | 2 | NR | 0 | 0 | NR | 0 | 0 | 0 | 2 | 2 | 0 |
| Silva (2014) | ^186^ | 0 | 0 | 2 | 0 | 2 | 0 | 0 | 0 | NR | 0 | 0 | NR | NR | NR | 0 | 0 | NR | 2 |
| Spencer (2015) | ^187^ | 2 | 0 | 2 | 0 | 2 | 2 | 0 | 2 | NR | 2 | 0 | NR | 0 | 2 | 2 | 2 | 2 | 2 |
| Tamayo (2010) | ^188^ | 0 | 0 | 2 | 0 | 0 | 0 | 0 | 0 | NR | 1 | 0 | NR | NR | NR | 0 | 2 | NR | 2 |
| Tang (2016) | ^189^ | 2 | 0 | 0 | 1 | 2 | 0 | 0 | 1 | NR | 1 | 0 | NR | 0 | 2 | 2 | 2 | 2 | 2 |
| Tatulashvili (2020) | ^190^ | 2 | 0 | 0 | 0 | 0 | 0 | 0 | 1 | NR | 0 | 0 | NR | NR | NR | 0 | 2 | NR | 2 |
| Thomson (2021) | ^191^ | 2 | 0 | 0 | 2 | 2 | 2 | 2 | 2 | NR | 1 | 0 | NR | 0 | 2 | 2 | 2 | 2 | 0 |
| Uphoff (2015) | ^192^ | 0 | 1 | 0 | 0 | 0 | 0 | 0 | 0 | NR | 0 | 2 | NR | 0 | 2 | 2 | 0 | 2 | 2 |
| Uthman (2013) | ^193^ | 2 | 0 | 2 | 0 | 2 | 2 | 0 | 0 | NR | 0 | 0 | NR | 2 | 0 | 0 | 2 | 2 | 2 |
| Valentin (2020) | ^194^ | 2 | 1 | 2 | 1 | 2 | 2 | 2 | 1 | NR | 1 | 0 | NR | 2 | 2 | 2 | 2 | 2 | 2 |
| Vart (2015) | ^195^ | 2 | 0 | 2 | 2 | 2 | 2 | 0 | 1 | NR | 1 | 0 | NR | 2 | 2 | 2 | 2 | 2 | 2 |
| Wagg (2021) | ^196^ | 2 | 1 | 0 | 0 | 2 | 2 | 2 | 2 | NR | 1 | 0 | NR | NR | NR | 2 | 2 | NR | 2 |
| Weightman (2012) | ^197^ | 2 | 0 | 2 | 2 | 0 | 2 | 0 | 2 | NR | 0 | 0 | NR | 2 | 0 | 0 | 0 | 2 | 2 |
| Williams (2018) | ^198^ | 2 | 0 | 2 | 1 | 0 | 0 | 0 | 1 | NR | 1 | 0 | NR | NR | NR | 2 | 2 | NR | 2 |
| Wojcicki (2005) | ^199^ | 0 | 0 | 0 | 0 | 0 | 0 | 2 | 0 | NR | 1 | 0 | NR | NR | NR | 2 | 2 | NR | 0 |
| Wu (2017) | ^200^ | 2 | 1 | 2 | 0 | 2 | 0 | 0 | 2 | NR | 2 | 0 | NR | NR | NR | 2 | 2 | NR | 2 |
| van Zwieten (2018) | ^201^ | 2 | 1 | 2 | 2 | 0 | 0 | 0 | 1 | NR | 2 | 0 | NR | NR | NR | 2 | 2 | NR | 2 |

*Abbreviations:* RCT – randomised controlled trials. NRS – non-randomised studies. *Notes:* NR – not relevant (because it was not a meta-analysis or no randomised controlled trials were included), 2 – Yes, 1 – Partial Yes, 0 – No. *Only applied to meta-analyses. #Only applied to reviews including randomised controlled trials (RCTs). AMSTAR 2 items are as follows: 1. Defined PICO question (Population, Intervention, Comparator, Outcome) 2. A priori methods in protocol 3. Justified inclusion of study designs 4. Comprehensive literature search 5. Duplicate study selection 6. Duplicate data extraction 7. Provided list of excluded studies and justification 8. Detailed description of included studies 9. Satisfactory risk of bias (ROB) assessment 10. Reported funding sources for included studies 11. Appropriate methods for meta-analysis 12. Assessed impact of ROB on meta-analysis results 13. Accounted for ROB when interpreting results 14. Explained and discussed any heterogeneity 15. Assessed publication bias 16. Review authors reported potential conflicts of interest including funding.

## Table S4. Approaches towards addressing overadjustment (OA) bias for included studies

| **First Author (Year)** | **Citation** | **Clearly defined E and O** | **Clearly defined C and M** | **Causal diagram** | **Included OA in ROB** | **Included confounding in ROB** | **SA related to OA^*^** | **Reported variables adjusted for in each study** | **Prioritised results from models with proper adjustment** | **Prioritised results from minimally adjusted models** | **Presented results with different levels of adjustment for comparison** | **Discussed OA in the text** | **Other approaches related to OA** |
| --- | --- | --- | --- | --- | --- | --- | --- | --- | --- | --- | --- | --- | --- |
| Adams (2018) | ^118^ | Yes | Somewhat | No | Unclear | Yes | No | No | Yes | No | No | No | None |
| Adegbosin (2019) | ^119^ | Yes | No | No | No | Yes | No | No | No | No | No | No | None |
| Amiresmaili (2018) | ^120^ | Yes | No | No | No | Yes | No | No | Unclear | Unclear | No | No | None |
| Amjad (2019) | ^121^ | Somewhat | No | No | No | Yes | No | No | No | No | Somewhat | No | None |
| Bastos (2011) | ^122^ | Yes | No | No | No | No | n/a | No | No | No | No | Yes | None |
| Best (2019) | ^123^ | Yes | Somewhat | No | Yes | Yes | No | Yes | No | No | Yes | Yes | None |
| Bijker (2016) | ^124^ | Somewhat | Somewhat | No | n/a - no ROB | n/a – no ROB | n/a | Yes | Unclear | No | No | No | None |
| Blumenshine (2010) | ^125^ | Yes | Somewhat | No | Unclear | Unclear | n/a | Somewhat | No | No | Somewhat | Yes | None |
| Boillot (2011) | ^126^ | Yes | Somewhat | No | n/a - no ROB | n/a - no ROB | No | Yes | No | No | Yes | No | None |
| Borschuk (2015) | ^127^ | Somewhat | No | No | n/a - no ROB | n/a - no ROB | n/a | No | No | No | No | No | For one study that considered mediation, the authors reported this in the results table. |
| Boss (2011) | ^128^ | No | No | No | n/a - no ROB | n/a - no ROB | n/a | No | No | No | No | No | None |
| Bougea (2022) | ^129^ | Yes | Somewhat | No | No | Yes | No | No | Yes | Unclear | No | No | None |
| Boylan (2018) | ^130^ | Yes | No | No | n/a - no ROB | n/a - no ROB | No | Yes | No | No | No | No | None |
| Bridger Staatz (2021) | ^131^ | Yes | Somewhat | No | No | Yes | n/a | Somewhat | No | No | Somewhat | No | Reported results of analyses in included studies that focused on mediation. |
| Bridger Staatz (2021) | ^132^ | Yes | Somewhat | No | No | Yes | n/a | No | No | No | Somewhat | Yes | None |
| Burneo (2009) | ^133^ | No | No | No | n/a - no ROB | n/a - no ROB | n/a | No | No | No | No | No | None |
| Chung (2016) | ^134^ | Yes | No | No | Unclear | Unclear | n/a | No | No | No | No | No | None |
| Conway (2008) | ^135^ | Yes | Somewhat | No | No | Yes | No | Yes | No | No | Yes | No | None |
| Costa (2018) | ^136^ | Somewhat | No | No | No | Yes | No | No | No | No | Yes | No | None |
| Crichton (2015) | ^137^ | Yes | No | No | No | Yes | No | Yes | No | No | No | Yes | None |
| Cundiff (2017) | ^138^ | Yes | No | No | n/a – no ROB | n/a – no ROB | Somewhat | Yes | No | No | Somewhat | No | None |
| Degarege (2019) | ^139^ | Yes | No | No | No | Yes | No | No | No | No | No | No | None |
| Didsbury (2016) | ^140^ | Yes | No | No | Unclear | Unclear | n/a | No | No | No | No | No | None |
| El-Sayed (2012) | ^141^ | Somewhat | No | No | n/a – no ROB | n/a – no ROB | n/a | Somewhat | No | No | Yes | No | None |
| El-Sayed (2012) | ^142^ | Yes | No | No | n/a – no ROB | n/a – no ROB | n/a | Yes | No | No | No | No | None |
| Elwadhi (2020) | ^143^ | Yes | No | No | No | Yes | n/a | Unclear | No | No | No | No | None |
| Etindele Sosso (2021) | ^144^ | Yes | Somewhat | No | No | Yes | n/a | No | No | No | No | No | None |
| Fruhstorfer (2016) | ^145^ | Somewhat | No | No | Unclear | Unclear | No | No | No | No | No | Somewhat | None |
| Fryers (2003) | ^146^ | Somewhat | No | No | n/a – no ROB | n/a – no ROB | n/a | No | No | No | No | No | None |
| Galobardes (2006) | ^147^ | Yes | No | No | n/a – no ROB | n/a – no ROB | n/a | No | No | No | Somewhat | Yes | None |
| Gershon (2012) | ^148^ | Somewhat | No | No | No | Yes | n/a | Yes | Unclear | Unclear | Somewhat | Yes | None |
| Goulden (2015) | ^149^ | Yes | No | No | No | Yes | n/a | Yes | No | No | No | Yes | None |
| Grintsova (2014) | ^150^ | Yes | No | No | No | Yes | n/a | Somewhat | No | No | No | No | None |
| Guglielmi (2019) | ^151^ | Somewhat | No | No | No | Yes | n/a | Somewhat | No | No | No | No | None |
| Hanna (2017) | ^152^ | No | No | No | No | No | n/a | No | No | No | Somewhat | No | None |
| Houweling (2016) | ^153^ | Somewhat | Yes | Yes | n/a – no ROB | n/a – no ROB | n/a | Yes | Yes | No | Yes | Yes | None |
| Karanth (2019) | ^154^ | Yes | No | No | n/a – no ROB | n/a – no ROB | No | No | No | No | No | No | None |
| Kim (2013) | ^155^ | Somewhat | Somewhat | Somewhat | n/a – no ROB | n/a – no ROB | n/a | Somewhat | No | No | No | No | None |
| Knorst (2021) | ^156^ | Somewhat | No | No | No | Yes | Somewhat | No | No | No | No | No | None |
| Kolahdooz (2015) | ^157^ | No | No | No | Unclear | Unclear | n/a | No | No | No | No | No | None |
| Lago (2018) | ^158^ | No | No | No | n/a – no ROB | n/a – no ROB | n/a | No | No | No | No | No | None |
| Lee (2021) | ^159^ | Yes | Somewhat | No | Unclear | Unclear | n/a | Somewhat | No | No | No | No | Reported results of analyses in included studies that focused on mediation. |
| Li (2021) | ^160^ | Yes | No | No | No | Yes | n/a | Yes | No | No | No | No | None |
| Lindner (2018) | ^161^ | Yes | No | No | No | Yes | n/a | Yes | No | No | No | No | None |
| Liu (2017) | ^162^ | Yes | Yes | Yes | n/a – no ROB | n/a – no ROB | No | Yes | Yes | Yes | Yes | Yes | Reported results of analyses in included studies that considered mediation by BMI or adult SES. |
| Lorant (2003) | ^163^ | Yes | Somewhat | No | No | Yes | No | No | No | No | Somewhat | No | None |
| Lund (2019) | ^164^ | Somewhat | No | No | Unclear | Unclear | n/a | No | No | No | No | No | None |
| Lundqvist (2016) | ^165^ | Yes | No | No | No | Yes | No | Yes | No | No | Yes | No | None |
| Lyle (2017) | ^166^ | Yes | No | No | Unclear | Unclear | n/a | No | No | No | No | No | None |
| Mahboob (2021) | ^167^ | Somewhat | No | No | No | Yes | n/a | No | No | No | No | No | None |
| Manrique-Garcia (2011) | ^168^ | Yes | Somewhat | No | n/a – no ROB | n/a – no ROB | Yes | Yes | No | No | Yes | Yes | None |
| Mihor (2020) | ^169^ | Yes | No | No | n/a – no ROB | n/a – no ROB | n/a | Yes | No | No | Yes | No | Reported results of analyses in included studies that focused on mediation. |
| Morton (2016) | ^170^ | Somewhat | Somewhat | No | Yes | Yes | n/a | Yes | Yes | No | No | Yes | None |
| Muscatell (2020) | ^171^ | Yes | Somewhat | No | n/a – no ROB | n/a – no ROB | Yes | Yes | No | Yes | Yes | Yes | None |
| O’Brien (2018) | ^172^ | Yes | Somewhat | No | No | Yes | n/a | No | No | No | No | No | None |
| Ombrellaro (2018) | ^173^ | Yes | Somewhat | No | No | Yes | Yes | No | Somewhat | No | No | No | None |
| Pan (2020) | ^174^ | Somewhat | No | No | No | Yes | No | No | No | No | No | No | None |
| Parikh (2003) | ^175^ | Yes | No | No | n/a – no ROB | n/a – no ROB | No | No | No | Yes | No | No | None |
| Petridou (2015) | ^176^ | Yes | Somewhat | No | No | Yes | No | Yes | No | No | No | No | None |
| Peykari (2015) | ^177^ | Yes | No | No | Unclear | Unclear | n/a | No | No | No | No | No | None |
| Pillas (2014) | ^178^ | Yes | No | No | n/a – no ROB | n/a – no ROB | n/a | No | No | No | No | No | None |
| Probst (2014) | ^179^ | Yes | Somewhat | No | No | Yes | No | Somewhat | No | Yes | No | Yes | None |
| Quon (2014) | ^180^ | Yes | No | No | No | Yes | Somewhat | No | Unclear | Unclear | Yes | No | None |
| Read (2016) | ^181^ | Yes | Somewhat | No | No | Somewhat | n/a | Somewhat | No | No | Somewhat | Yes | Reported results of analyses in included studies that focused on mediation. |
| Schwendicke (2015) | ^182^ | Yes | Somewhat | No | No | Yes | No | No | Yes | No | No | No | None |
| Scott (2017) | ^183^ | Yes | Somewhat | No | No | Yes | n/a | No | No | No | Yes | No | None |
| Senese (2009) | ^184^ | Yes | Somewhat | No | n/a – no ROB | n/a – no ROB | n/a | Yes | Yes | No | Yes | No | None |
| Sidorchuk (2009) | ^185^ | Yes | No | No | n/a – no ROB | n/a – no ROB | No | Yes | No | No | Yes | No | None |
| Silva (2014) | ^186^ | Somewhat | No | No | No | Yes | n/a | No | No | No | No | No | None |
| Spencer (2015) | ^187^ | Yes | Somewhat | No | No | Yes | Somewhat | Yes | No | No | Yes | Yes | None |
| Tamayo (2010) | ^188^ | Yes | Somewhat | No | No | Yes | n/a | Somewhat | No | No | Somewhat | Somewhat | None |
| Tang (2016) | ^189^ | Yes | No | No | No | Yes | Yes | No | No | No | Yes | Yes | None |
| Tatulashvili (2020) | ^190^ | Yes | Somewhat | No | n/a – no ROB | n/a – no ROB | n/a | Yes | No | No | Yes | No | Reported results of analyses in included studies that focused on mediation, particularly for glycemic control. |
| Thomson (2021) | ^191^ | Yes | No | No | No | Yes | No | Yes | No | No | Somewhat | No | None |
| Uphoff (2015) | ^192^ | Somewhat | No | No | No | Yes | No | Yes | No | No | Yes | No | None |
| Uthman (2013) | ^193^ | Yes | No | No | n/a – no ROB | n/a – no ROB | Yes | No | No | No | Yes | Yes | None |
| Valentin (2020) | ^194^ | Yes | No | No | No | Yes | No | No | Yes | No | No | Yes | Risk of bias included consideration of whether information was provided on variables included in multivariable analysis. Discussed findings from studies that considered mediation. |
| Vart (2015) | ^195^ | Yes | No | No | No | Yes | Yes | Somewhat | Yes | No | Somewhat | Yes | None |
| Wagg (2021) | ^196^ | Yes | Somewhat | No | No | Yes | n/a | Yes | No | No | No | Yes | None |
| Weightman (2012) | ^197^ | Yes | No | No | Unclear | Unclear | No | Somewhat | No | No | Yes | No | None |
| Williams (2018) | ^198^ | Yes | No | No | No | Yes | n/a | Somewhat | No | No | Yes | No | None |
| Wojcicki (2005) | ^199^ | Yes | Yes | No | Somewhat | Somewhat | n/a | Somewhat | Yes | No | Yes | Yes | None |
| Wu (2017) | ^200^ | Yes | Somewhat | No | No | Yes | n/a | Yes | Unclear | Unclear | No | Yes | None |
| van Zwieten (2018) | ^201^ | Yes | Yes | No | No | Yes | n/a | Yes | Yes | No | No | No | None |

*Abbreviations.* E: exposure(s). O: outcome(s). C: confounder(s). M: mediator(s). ROB: risk of bias. OA: overadjustment. n/a: not applicable. *Notes:* ^*^Only applied to meta-analyses.

## Figure S1. Number of approaches to overadjustment applied by included studies

*Figure S1a. Number of approaches applied in systematic reviews (out of a maximum of 11 approaches). N=47 systematic reviews included*

*Figure S1b. Number of approaches applied in meta-analyses (out of a maximum of 12 approaches). N=37 meta-analyses included.*

**References**

1. Gebremariam MK, Lien N, Nianogo RA, Arah OA. Mediators of socioeconomic differences in adiposity among youth: a systematic review. *Obes Rev* 2017; **18**: 880-98.

2. Hoven H, Siegrist J. Work characteristics, socioeconomic position and health: a systematic review of mediation and moderation effects in prospective studies. *Occup Environ Med* 2013; **70**: 663-9.

3. Kerr GD, Slavin H, Clark D, Coupar F, Langhorne P, Stott DJ. Do vascular risk factors explain the association between socioeconomic status and stroke incidence: a meta-analysis. *Cerebrovasc Dis* 2011; **31**: 57-63.

4. Kroger H, Pakpahan E, Hoffmann R. What causes health inequality? A systematic review on the relative importance of social causation and health selection. *Eur J Public Health* 2015; **25**: 951-60.

5. Lee C-Y, Lee Y-H. Measurement of Socioeconomic Position in Research on Cardiovascular Health Disparities in Korea: A Systematic Review. *J Prev Med Public Health* 2019; **52**: 281-91.

6. Mech P, Hooley M, Skouteris H, Williams J. Parent-related mechanisms underlying the social gradient of childhood overweight and obesity: a systematic review. *Child Care Health Dev* 2016; **42**: 603-24.

7. Probst C, Kilian C, Sanchez S, Lange S, Rehm J. The role of alcohol use and drinking patterns in socioeconomic inequalities in mortality: a systematic review. *Lancet Public Health* 2020; **5**: e324-e32.

8. Aljassim N, Ostini R. Health literacy in rural and urban populations: A systematic review. *Patient Educ Couns* 2020; **103**: 2142-54.

9. Almeida APSC, Nunes BP, Duro SMS, Facchini LA. Socioeconomic determinants of access to health services among older adults: a systematic review. *Rev Saude Publica* 2017; **51**: 50.

10. Asafu-Adjei D, Gu M, Pagano M, Onyeji I, Stahl P. Socioeconomic disparities in the treatment of erectile dysfunction: A systematic review. *Andrology* 2017; **5 (Supplement 1)**: 93-4.

11. Bocquier A, Ward J, Raude J, Peretti-Watel P, Verger P. Socioeconomic differences in childhood vaccination in developed countries: a systematic review of quantitative studies. *Expert Rev Vaccines* 2017; **16**: 1107-18.

12. Casetta B, Videla AJ, Bardach A, et al. Association Between Cigarette Smoking Prevalence and Income Level: A Systematic Review and Meta-Analysis. *Nicotine Tob Res* 2017; **19**: 1401-7.

13. Corrigan KL, Wall KC, Bartlett JA, Suneja G. Cancer disparities in people with HIV: A systematic review of screening for non-AIDS-defining malignancies. *Cancer* 2019; **125**: 843-53.

14. Davies JM, Sleeman KE, Leniz J, et al. Socioeconomic position and use of healthcare in the last year of life: A systematic review and meta-analysis. *PLoS medicine* 2019; **16**: e1002782.

15. Eilenberg JS, Paff M, Harrison AJ, Long KA. Disparities Based on Race, Ethnicity, and Socioeconomic Status Over the Transition to Adulthood Among Adolescents and Young Adults on the Autism Spectrum: a Systematic Review. *Curr Psychiatry Rep* 2019; **21**: 32.

16. Forrest LF, Adams J, Wareham H, Rubin G, White M. Socioeconomic inequalities in lung cancer treatment: systematic review and meta-analysis. *PLoS medicine* 2013; **10**: e1001376.

17. Forrest LF, Sowden S, Rubin G, White M, Adams J. Socio-economic inequalities in patient, primary care, referral, diagnostic, and treatment intervals on the lung cancer care pathway: protocol for a systematic review and meta-analysis. *Syst Rev* 2014; **3**: 30.

18. Forrest LF, Sowden S, Rubin G, White M, Adams J. Socio-economic inequalities in stage at diagnosis, and in time intervals on the lung cancer pathway from first symptom to treatment: systematic review and meta-analysis. *Thorax* 2017; **72**: 430-6.

19. Frederiksen L, Mader L, Feychting M, et al. Surviving childhood cancer: A systematic review on risk and determinants of adverse socioeconomic outcomes. *Pediatr Blood Cancer* 2018; **65 (Supplement 2)**: S683-S4.

20. Frier A, Barnett F, Devine S. The relationship between social determinants of health, and rehabilitation of neurological conditions: a systematic literature review. *Disabil Rehabil* 2017; **39**: 941-8.

21. Giskes K, Avendano M, Brug J, Kunst AE. A systematic review of studies on socioeconomic inequalities in dietary intakes associated with weight gain and overweight/obesity conducted among European adults. *Obes Rev* 2010; **11**: 413-29.

22. Hyun KK, Brieger D, Woodward M, Richtering S, Redfern J. The effect of socioeconomic disadvantage on prescription of guideline-recommended medications for patients with acute coronary syndrome: systematic review and meta-analysis. *Int J Equity Health* 2017; **16**: 162.

23. Jain A, van Hoek AJ, Boccia D, Thomas SL. Lower vaccine uptake amongst older individuals living alone: A systematic review and meta-analysis of social determinants of vaccine uptake. *Vaccine* 2017; **35**: 2315-28.

24. Konradsen AA, Lund CM, Vistisen KK, Albieri V, Dalton SO, Nielsen DL. The influence of socioeconomic position on adjuvant treatment of stage III colon cancer: a systematic review and meta-analysis. *Acta Oncol* 2020; **59**: 1291-9.

25. Langlois EV, Miszkurka M, Zunzunegui MV, Ghaffar A, Ziegler D, Karp I. Inequities in postnatal care in low- and middle-income countries: a systematic review and meta-analysis. *Bull World Health Organ* 2015; **93**: 259-70G.

26. Mayen AL, Marques-Vidal P, Paccaud F, Bovet P, Stringhini S. Socioeconomic determinants of dietary patterns in low- and middle-income countries: a systematic review. *Am J Clin Nutr* 2014; **100**: 1520-31.

27. Mekonnen T, Havdal HH, Lien N, et al. Mediators of socioeconomic inequalities in dietary behaviours among youth: A systematic review. *Obes Rev* 2020; **21**: e13016.

28. Nagata JM, Hernandez-Ramos I, Kurup AS, Albrecht D, Vivas-Torrealba C, Franco-Paredes C. Social determinants of health and seasonal influenza vaccination in adults >=65 years: a systematic review of qualitative and quantitative data. *BMC Public Health* 2013; **13**: 388.

29. Neergaard MA, Brunoe AH, Skorstengaard MH, Nielsen MK. What socio-economic factors determine place of death for people with life-limiting illness? A systematic review and appraisal of methodological rigour. *Palliat Med* 2019; **33**: 900-25.

30. Reda SF, Reda SM, Thomson WM, Schwendicke F. Inequality in Utilization of Dental Services: A Systematic Review and Meta-analysis. *Am J Public Health* 2018; **108**: e1-e7.

31. Schroder SL, Richter M, Schroder J, Frantz S, Fink A. Socioeconomic inequalities in access to treatment for coronary heart disease: A systematic review. *Int J Cardiol* 2016; **219**: 70-8.

32. Thobie A, Mulliri A, Bouvier V, Launoy G, Alves A, Dejardin O. Same Chance of Accessing Resection? Impact of Socioeconomic Status on Resection Rates Among Patients with Pancreatic Adenocarcinoma-A Systematic Review. *Health Equity* 2021; **5**: 143-50.

33. Tromp N, Michels C, Mikkelsen E, Hontelez J, Baltussen R. Equity in utilization of antiretroviral therapy for HIV-infected people in South Africa: a systematic review. *Int J Equity Health* 2014; **13**: 60.

34. Wallar LE, De Prophetis E, Rosella LC. Socioeconomic inequalities in hospitalizations for chronic ambulatory care sensitive conditions: a systematic review of peer-reviewed literature, 1990-2018. *Int J Equity Health* 2020; **19**: 60.

35. Zarnowiecki DM, Dollman J, Parletta N. Associations between predictors of children's dietary intake and socioeconomic position: a systematic review of the literature. *Obes Rev* 2014; **15**: 375-91.

36. Abbott LS, Elliott LT. Eliminating Health Disparities through Action on the Social Determinants of Health: A Systematic Review of Home Visiting in the United States, 2005-2015. *Public Health Nurs* 2017; **34**: 2-30.

37. Afshar N, English DR, Milne RL. Rural-urban residence and cancer survival in high-income countries: A systematic review. *Cancer* 2019; **125**: 2172-84.

38. Alston L, Allender S, Peterson K, Jacobs J, Nichols M. Rural Inequalities in the Australian Burden of Ischaemic Heart Disease: A Systematic Review. *Heart Lung Circ* 2017; **26**: 122-33.

39. Andrea SB, Hooker ER, Messer LC, Tandy T, Boone-Heinonen J. Does the association between early life growth and later obesity differ by race/ethnicity or socioeconomic status? A systematic review. *Ann Epidemiol* 2017; **27**: 583-92.e5.

40. Anonymous. Correction to: Income inequality and depression: a systematic review and meta-analysis of the association and a scoping review of mechanisms (World Psychiatry, (2018), 17, 1, (76-89), 10.1002/wps.20492). *World Psychiatry* 2018; **17**: 235.

41. Assaf S, Juan C. Stunting and Anemia in Children from Urban Poor Environments in 28 Low and Middle-income Countries: A Meta-analysis of Demographic and Health Survey Data. *Nutrients* 2020; **12**: 3539.

42. Burns JK, Tomita A, Kapadia AS. Income inequality and schizophrenia: increased schizophrenia incidence in countries with high levels of income inequality. *Int J Soc Psychiatry* 2014; **60**: 185-96.

43. Cairns JM, Graham E, Bambra C. Area-level socioeconomic disadvantage and suicidal behaviour in Europe: A systematic review. *Soc Sci Med* 2017; **192**: 102-11.

44. Chan CQH, Lee KH, Low LL. A systematic review of health status, health seeking behaviour and healthcare utilisation of low socioeconomic status populations in urban Singapore. *Int J Equity Health* 2018; **17**: 39.

45. Christiani Y, Dhippayom T, Chaiyakunapruk N. Assessing evidence of inequalities in access to medication for diabetic populations in low- and middle-income countries: a systematic review. *Glob Health Action* 2016; **9**: 32505.

46. Cullati S, Rousseaux E, Gabadinho A, Courvoisier DS, Burton-Jeangros C. Factors of change and cumulative factors in self-rated health trajectories: a systematic review. *Adv Life Course Res* 2014; **19**: 14-27.

47. Harding K, Mersha TB, Pham PT, et al. Health Disparities in Kidney Transplantation for African Americans. *Am J Nephrol* 2017; **46**: 165-75.

48. Ireland MJ, March S, Crawford-Williams F, et al. A systematic review of geographical differences in management and outcomes for colorectal cancer in Australia. *BMC Cancer* 2017; **17**: 95.

49. Johnson JAI, Johnson AM. Urban-rural differences in childhood and adolescent obesity in the United States: a systematic review and meta-analysis. *Child Obes* 2015; **11**: 233-41.

50. Lynch J, Smith GD, Harper S, et al. Is income inequality a determinant of population health? Part 1. A systematic review. *The Milbank quarterly* 2004; **82**: 5-99.

51. Mello D, Wiebe D. The Role of Socioeconomic Status in Latino Health Disparities Among Youth with Type 1 Diabetes: a Systematic Review. *Curr Diab Rep* 2020; **20**: 56.

52. Patel V, Burns JK, Dhingra M, Tarver L, Kohrt BA, Lund C. Income inequality and depression: a systematic review and meta-analysis of the association and a scoping review of mechanisms. *World Psychiatry* 2018; **17**: 76-89.

53. Ribeiro WS, Bauer A, Andrade MCR, et al. Income inequality and mental illness-related morbidity and resilience: a systematic review and meta-analysis. *Lancet Psychiatry* 2017; **4**: 554-62.

54. Russell CG, Taki S, Laws R, et al. Effects of parent and child behaviours on overweight and obesity in infants and young children from disadvantaged backgrounds: systematic review with narrative synthesis. *BMC Public Health* 2016; **16**: 151.

55. Coughlin SS. Social determinants of breast cancer risk, stage, and survival. *Breast Cancer Res Treat* 2019; **177**: 537-48.

56. Coughlin SS, Young L. Social Determinants of Myocardial Infarction Risk and Survival: A Systematic Review. *Eur j Cardiovasc Res* 2020; **1**.

57. Galvin A, Delva F, Helmer C, et al. Sociodemographic, socioeconomic, and clinical determinants of survival in patients with cancer: A systematic review of the literature focused on the elderly. *J Geriatr Oncol* 2018; **9**: 6-14.

58. LeBrun DG, Banskota B, Banskota AK, Rajbhandari T, Baldwin KD, Spiegel DA. Socioeconomic Status Influences Functional Severity of Untreated Cerebral Palsy in Nepal: A Prospective Analysis and Systematic Review. *Clin Orthop Relat Res* 2019; **477**: 10-21.

59. Vainshtein J. Disparities in breast cancer incidence across racial/ethnic strata and socioeconomic status: a systematic review. *J Natl Med Assoc* 2008; **100**: 833-9.

60. Adewuyi EO, Auta A. Medical injection and access to sterile injection equipment in low- and middle-income countries: a meta-analysis of Demographic and Health Surveys (2010-2017). *Int Health* 2020; **12**: 388-94.

61. Almeida LM, Caldas J, Ayres-de-Campos D, Salcedo-Barrientos D, Dias S. Maternal healthcare in migrants: a systematic review. *Matern Child Health J* 2013; **17**: 1346-54.

62. Alshamsan R, Majeed A, Ashworth M, Car J, Millett C. Impact of pay for performance on inequalities in health care: systematic review. *J Health Serv Res Policy* 2010; **15**: 178-84.

63. Apor A, Pagaling GT, Espiritu AI, Jamora RDG. Stroke Research Disparity in Southeast Asia: Socioeconomic Factors, Healthcare Delivery, and Stroke Disease Burden. *J Stroke Cerebrovasc Dis* 2021; **30**: 105481.

64. Awoh AB, Plugge E. Immunisation coverage in rural-urban migrant children in low and middle-income countries (LMICs): a systematic review and meta-analysis. *J Epidemiol Community Health* 2016; **70**: 305-11.

65. Backholer K, Gupta A, Zorbas C, et al. Differential exposure to, and potential impact of, unhealthy advertising to children by socio-economic and ethnic groups: A systematic review of the evidence. *Obes Rev* 2021; **22**: e13144.

66. Demetrio F, Teles CAS, Santos DBD, Pereira M. Food insecurity in pregnant women is associated with social determinants and nutritional outcomes: a systematic review and meta-analysis. *Cien Saude Colet* 2020; **25**: 2663-76.

67. Lu JB, Danko KJ, Elfassy MD, Welch V, Grimshaw JM, Ivers NM. Do quality improvement initiatives for diabetes care address social inequities? Secondary analysis of a systematic review. *BMJ Open* 2018; **8**: e018826.

68. Abbott LS, Williams CL. Influences of Social Determinants of Health on African Americans Living With HIV in the Rural Southeast: A Qualitative Meta-synthesis. *J Assoc Nurses AIDS Care* 2015; **26**: 340-56.

69. Arsenault C, Harper S, Nandi A, Mendoza Rodriguez JM, Hansen PM, Johri M. Monitoring equity in vaccination coverage: A systematic analysis of demographic and health surveys from 45 Gavi-supported countries. *Vaccine* 2017; **35**: 951-9.

70. Arsenault C, Jordan K, Lee D, et al. Equity in antenatal care quality: an analysis of 91 national household surveys. *Lancet Glob Health* 2018; **6**: e1186-e95.

71. Askari A, Aziz O, Currie A, Nachiappan S, Athanasiou T, Faiz O. Inequalities in colorectal cancer risk and educational level in developed countries: A systematic review and meta-analysis of observational studies. *Colorectal Dis* 2014; **16 (Supplement 2)**: 77.

72. Attree P. Low-income mothers, nutrition and health: a systematic review of qualitative evidence. *Matern Child Nutr* 2005; **1**: 227-40.

73. Bambra CL, Hillier FC, Moore HJ, Summerbell CD. Tackling inequalities in obesity: a protocol for a systematic review of the effectiveness of public health interventions at reducing socioeconomic inequalities in obesity amongst children. *Syst Rev* 2012; **1**: 16.

74. Bird S, Talevski J, Feehan J, et al. Associations between household income and proinflammatory cytokines related to bone early in the life-course: A Systematic Review. *J Bone Miner Res* 2020; **35 (SUPPL 1)**: 94.

75. Boutayeb A, Boutayeb S, Boutayeb W. Multi-morbidity of non communicable diseases and equity in WHO Eastern Mediterranean countries. *Int J Equity Health* 2013; **12**: 60.

76. Bridger Staatz C, Blodgett J, George A, Hardy R. Socioeconomic position and lifetime body composition: A systematic review. *Obes Facts* 2019; **12 (Supplement 1)**: 235-6.

77. Brinda EM, Rajkumar AP, Attermann J, Gerdtham UG, Enemark U, Jacob KS. Health, Social, and Economic Variables Associated with Depression Among Older People in Low and Middle Income Countries: World Health Organization Study on Global AGEing and Adult Health. *Am J Geriatr Psychiatry* 2016; **24**: 1196-208.

78. Burch R, Rizzoli P, Loder E. The prevalence and impact of migraine and severe headache in the United States: Updated age, sex, and socioeconomic-specific estimates from government health surveys. *Headache* 2021; **61**: 60-8.

79. Chung AC, Backholer K, Wong E, Palermo C, Keating C, Peeters A. Trends in childhood obesity prevalence according to socioeconomic position: A systematic review. *Obes Res Clin Pract* 2014; **8 (Supplement 1)**: 18.

80. Claassen MA, Papies EK, Hardman CA, Robinson E. Socioeconomic differences in the susceptibility to overeat from excessive portions of unhealthy food. *Obes Facts* 2019; **12 (Supplement 1)**: 30.

81. Coylewright M, Branda M, Inselman JW, et al. Impact of sociodemographic patient characteristics on the efficacy of decision AIDS: a patient-level meta-analysis of 7 randomized trials. *Circ Cardiovasc Qual Outcomes* 2014; **7**: 360-7.

82. Davies JM, Maddocks M, Sleeman KE, Martelli Leniz J, Wilson R, Murtagh FEM. Associations between different area-based and individual-level measures of socioeconomic position (SEP) and health outcomes in the last year of life: A systematic review. *Palliat Med* 2018; **32 (1 Supplement 1)**: 197.

83. de Mestral C, Stringhini S. Socioeconomic Status and Cardiovascular Disease: an Update. *Curr Cardiol Rep* 2017; **19**: 115.

84. Dobson KG, Vigod SN, Mustard C, Smith PM. Trends in the prevalence of depression and anxiety disorders among Canadian working-age adults between 2000 and 2016. *Health Rep* 2020; **31**: 12-23.

85. Dos Santos MA. Unemployment, mental disorders and suicide: A systematic review. *Eur J Epidemiol* 2015; **30**: 984.

86. Elani HW, Harper S, Thomson WM, et al. Social inequalities in tooth loss: A multinational comparison. *Community Dent Oral Epidemiol* 2017; **45**: 266-74.

87. Gershon AS, Dolmage TE, Stephenson A. Socioeconomic status (SES) and Chronic Obstructive Pulmonary Disease (COPD): A systematic literature review. *Am J Respir Crit Care Med* 2011; **183**: A1486.

88. Griffiths KE, Young J. Disparities in lung cancer: A systematic literature review. *Asia Pac J Clin Oncol* 2011; **7 (Supplement 4)**: 168.

89. Grittner U, Kuntsche S, Graham K, Bloomfield K. Social inequalities and gender differences in the experience of alcohol-related problems. *Alcohol Alcohol* 2012; **47**: 597-605.

90. Guglielmi O, Lanteri P, Garbarino S. Association between sleep disordered breathing symptoms, sleep apnea and socioeconomic status: A systematic review of the literature. *J Sleep Res* 2018; **27 (Supplement 1)**: 286-7.

91. Guglielmi O, Lanteri P, Garbarino S. Sleep duration and socio-economic status in pediatric population: A systematic review of the literature. *J Sleep Res* 2018; **27 (Supplement 1)**: 347.

92. Hajizadeh M, Nandi A, Heymann J. Social inequality in infant mortality: what explains variation across low and middle income countries? *Soc Sci Med* 2014; **101**: 36-46.

93. Intzes S, Symeonidou M, Zagoridis K, et al. Socioeconomic Status is Globally a Prognostic Factor for Overall Survival of Multiple Myeloma Patients: Synthesis of Studies and Review of the Literature. *Mediterr J Hematol Infect Dis* 2021; **13**: e2021006.

94. Jamaludin M, Nazar GP, Palladino R, Tsakos G, Watt RG, Millett C. Smoke-free legislation and socioeconomic inequalities in smoking-related morbidity and mortality among adults: A systematic review. *Tob Induc Dis* 2018; **16 (Supplement 1)**: A388.

95. Koenig JT, Busch MA. Trends in social inequalities in cardiovascular disease among the general population in high-income countries: A systematic review. *Eur J Prev Cardiol* 2017; **24 (Supplement 1)**: S148.

96. Kondo N. Socioeconomic disparities and health: impacts and pathways. *J Epidemiol* 2012; **22**: 2-6.

97. Kourti M, Sergentanis T, Perlepe C, et al. Do parental occupation and area remoteness lead to social disparities in survival from childhood leukemia? Re-analysis of databases and a meta-analysis. *Pediatr Blood Cancer* 2014; **61 (Supplement 2)**: S173-S4.

98. Lebrun DG, Banskota B, Banskota AK, Rajbhandari T, Baldwin KD, Spiegel DA. Erratum to: Socioeconomic status influences functional severity of untreated cerebral palsy in Nepal: A prospective analysis and systematic review (Clinical Orthopaedics and Related Research DOI: 10.1097/CORR.0000000000000476). *Clin Orthop Relat Res* 2019; **477**: 262-3.

99. Linder A, Gerdtham UG, Trygg N, Fritzell S, Saha S. Inequalities in the economic consequences of depression and anxiety in Europe: a systematic scoping review. *Eur J Public Health* 2020; **30**: 767-77.

100. Mamelund SE, Shelley-Egan C, Rogeberg O. The association between socioeconomic status and pandemic influenza: protocol for a systematic review and meta-analysis. *Syst Rev* 2019; **8**: 5.

101. McKenzie F, Zietsman A, Galukande M, et al. Breast cancer awareness in the sub-Saharan African ABC-DO cohort: African Breast Cancer-Disparities in Outcomes study. *Cancer Causes Control* 2018; **29**: 721-30.

102. Mejia GC, Elani HW, Harper S, et al. Socioeconomic status, oral health and dental disease in Australia, Canada, New Zealand and the United States. *BMC Oral Health* 2018; **18**: 176.

103. Purser A, Farinas A, Cruz C, Postoev A, Ibikunle C, Sanni A. Outcomes of bariatric surgery in patients of lower socioeconomic status versus patients of higher socioeconomic status. *Surg Endosc* 2016; **30 (Supplement 1)**: S443.

104. Rapp L, Sourdet S, Vellas B, Lacoste-Ferre MH. Oral Health and the Frail Elderly. *J Frailty Aging* 2017; **6**: 154-60.

105. Rose TC, Adams N, Taylor-Robinson DC, et al. Relationship between socioeconomic status and gastrointestinal infections in developed countries: a systematic review protocol. *Syst Rev* 2016; **5**: 13.

106. Rottmann N, Johansen C. Chronically ill patients in socially disadvantaged populations: A systematic review of interventions targeting health disparities in cancer, diabetes, and cardiovascular diseases. *Psychooncology* 2009; **18 (Supplement 2)**: S56-S7.

107. Rydland HT, Fjaer EL, Eikemo TA, et al. Educational inequalities in mortality amenable to healthcare. A comparison of European healthcare systems. *PLoS ONE* 2020; **15**: e0234135.

108. Singh A, Peres MA, Watt RG. The Relationship between Income and Oral Health: A Critical Review. *J Dent Res* 2019; **98**: 853-60.

109. Taib BG, Rylands J, Povall S, Jones TM, Taylor-Robinson D. Protocol: systematic review of the association between socio-economic status and survival in adult head and neck cancer. *Syst Rev* 2017; **6**: 151.

110. Valero-Elizondo J, Spatz ES, Salami JA, et al. Persistent SES disparities in cardiovascular risk factors and health in the United States: MEPS 2002-2013. *Circ Cardiovasc Qual Outcomes* 2017; **10 (Supplement 3)**: A254.

111. Vazquez CE, Cubbin C. Socioeconomic Status and Childhood Obesity: a Review of Literature from the Past Decade to Inform Intervention Research. *Curr Obes Rep* 2020; **9**: 562-70.

112. Wang Y, Beydoun MA. The obesity epidemic in the United States--gender, age, socioeconomic, racial/ethnic, and geographic characteristics: a systematic review and meta-regression analysis. *Epidemiol Rev* 2007; **29**: 6-28.

113. Wardle RA, Wardle AJ, Charadva C, Ghosh S, Moran GW. Literature review: Impacts of socioeconomic status on the risk of inflammatory bowel disease and its outcomes. *Eur J Gastroenterol Hepatol* 2017; **29**: 879-84.

114. Xu R, Zhao Q, Coelho M, et al. Socioeconomic inequality in vulnerability to all-cause and cause-specific hospitalisation associated with temperature variability: a time-series study in 1814 Brazilian cities. *Lancet Planet Health* 2020; **4**: e566-e76.

115. Zhou W, Chen R, Hopkins A. Association between socioeconomic status and incident stroke in China. *Int J Stroke* 2020; **15 (Supplement 1)**: 103.

116. Lindner LME, Rathmann W, Rosenbauer J. Inequalities in glycaemic control, hypoglycaemia and diabetic ketoacidosis according to socio-economic status and area-level deprivation in Type 1 diabetes mellitus: a systematic review. *Diabet Med* 2018b; **35**: 12-32.

117. Tatulashvili S, Fagherazzi G, Dow C, Cohen R, Fosse S, Bihan H. Socioeconomic inequalities and type 2 diabetes complications: A systematic review. *Diabetes Metab* 2020b; **46**: 89-99.

118. Adams NL, Rose TC, Hawker J, et al. Relationship between socioeconomic status and gastrointestinal infections in developed countries: A systematic review and meta-analysis. *PLoS ONE* 2018; **13**: e0191633.

119. Adegbosin AE, Zhou H, Wang S, Stantic B, Sun J. Systematic review and meta-analysis of the association between dimensions of inequality and a selection of indicators of Reproductive, Maternal, Newborn and Child Health (RMNCH). *J Glob Health* 2019; **9**: 010429.

120. Amiresmaili M, Amini S, Shahravan A, et al. Relation between Socioeconomic Indicators and Children Dental Caries in Iran: A Systematic Review and Meta-analysis. *Int J Prev Med* 2018; **9**: 71.

121. Amjad S, MacDonald I, Chambers T, et al. Social determinants of health and adverse maternal and birth outcomes in adolescent pregnancies: A systematic review and meta-analysis. *Paediatr Perinat Epidemiol* 2019; **33**: 88-99.

122. Bastos JL, Boing AF, Peres KG, Antunes JLF, Peres MA. Periodontal outcomes and social, racial and gender inequalities in Brazil: A systematic review of the literature between 1999 and 2008. *Cad Saude Publica* 2011; **27**: 141-53.

123. Best KE, Vieira R, Glinianaia SV, Rankin J. Socio-economic inequalities in mortality in children with congenital heart disease: A systematic review and meta-analysis. *Paediatr Perinat Epidemiol* 2019; **33**: 291-309.

124. Bijker R, Agyemang C. The influence of early-life conditions on cardiovascular disease later in life among ethnic minority populations: a systematic review. *Intern Emerg Med* 2016; **11**: 341-53.

125. Blumenshine P, Egerter S, Barclay CJ, Cubbin C, Braveman PA. Socioeconomic Disparities in Adverse Birth Outcomes: A Systematic Review. *Am J Prev Med* 2010; **39**: 263-72.

126. Boillot A, El Halabi B, Batty GD, Range H, Czernichow S, Bouchard P. Education as a predictor of chronic periodontitis: a systematic review with meta-analysis population-based studies. *PLoS ONE* 2011; **6**: e21508.

127. Borschuk AP, Everhart RS. Health disparities among youth with type 1 diabetes: A systematic review of the current literature. *Fam Syst Health* 2015; **33**: 297-313.

128. Boss EF, Smith DF, Ishman SL. Racial/ethnic and socioeconomic disparities in the diagnosis and treatment of sleep-disordered breathing in children. *Int J Pediatr Otorhinolaryngol* 2011; **75**: 299-307.

129. Bougea A, Anagnostouli M, Angelopoulou E, Spanou I, Chrousos G. Psychosocial and Trauma-Related Stress and Risk of Dementia: A Meta-Analytic Systematic Review of Longitudinal Studies. *J Geriatr Psychiatry Neurol* 2022; **35**: 24-37.

130. Boylan JM, Cundiff JM, Matthews KA. Socioeconomic Status and Cardiovascular Responses to Standardized Stressors: A Systematic Review and Meta-Analysis. *Psychosom Med* 2018; **80**: 278-93.

131. Bridger Staatz C, Kelly Y, Lacey RE, et al. Life course socioeconomic position and body composition in adulthood: a systematic review and narrative synthesis. *Int J Obes* 2021; **45**: 2300-15.

132. Bridger Staatz C, Kelly Y, Lacey RE, et al. Socioeconomic position and body composition in childhood in high- and middle-income countries: a systematic review and narrative synthesis. *Int J Obes* 2021; **45**: 2316-34.

133. Burneo JG, Jette N, Theodore W, et al. Disparities in epilepsy: report of a systematic review by the North American Commission of the International League Against Epilepsy. *Epilepsia* 2009; **50**: 2285-95.

134. Chung A, Backholer K, Wong E, Palermo C, Keating C, Peeters A. Trends in child and adolescent obesity prevalence in economically advanced countries according to socioeconomic position: a systematic review: Child obesity trends and socio-economic position. *Obes Rev* 2016; **17**: 276-95.

135. Conway DI, Petticrew M, Marlborough H, Berthiller J, Hashibe M, Macpherson LM. Socioeconomic inequalities and oral cancer risk: a systematic review and meta-analysis of case-control studies. *Int J Cancer* 2008; **122**: 2811-9.

136. Costa SM, Martins CC, Pinto MQC, Vasconcelos M, Abreu M. Socioeconomic Factors and Caries in People between 19 and 60 Years of Age: An Update of a Systematic Review and Meta-Analysis of Observational Studies. *Int J Environ Res Public Health* 2018; **15**: 18.

137. Crichton J, Hickman M, Campbell R, Batista-Ferrer H, Macleod J. Socioeconomic factors and other sources of variation in the prevalence of genital chlamydia infections: A systematic review and meta-analysis. *BMC Public Health* 2015; **15**: 729.

138. Cundiff JM, Matthews KA. Is subjective social status a unique correlate of physical health? A meta-analysis. *Health Psychol* 2017; **36**: 1109-25.

139. Degarege A, Fennie K, Degarege D, Chennupati S, Madhivanan P. Improving socioeconomic status may reduce the burden of malaria in sub Saharan Africa: A systematic review and meta-analysis. *PLoS ONE* 2019; **14**: e0211205.

140. Didsbury MS, Kim S, Medway MM, et al. Socio-economic status and quality of life in children with chronic disease: A systematic review. *J Paediatr Child Health* 2016; **52**: 1062-9.

141. El-Sayed AM, Scarborough P, Galea S. Socioeconomic inequalities in childhood obesity in the United Kingdom: a systematic review of the literature. *Obes Facts* 2012; **5**: 671-92.

142. El-Sayed AM, Scarborough P, Galea S. Unevenly distributed: a systematic review of the health literature about socioeconomic inequalities in adult obesity in the United Kingdom. *BMC Public Health* 2012; **12**: 18.

143. Elwadhi D, Cohen A. Social inequalities in antidepressant treatment outcomes: a systematic review. *Soc Psychiatry Psychiatr Epidemiol* 2020; **55**: 1241-59.

144. Etindele Sosso FA, Matos E. Socioeconomic disparities in obstructive sleep apnea: a systematic review of empirical research. *Sleep Breath* 2021; **25**: 1729-39.

145. Fruhstorfer BH, Mousoulis C, Uthman OA, Robertson W. Socio-economic status and overweight or obesity among school-age children in sub-Saharan Africa - a systematic review. *Clin Obes* 2016; **6**: 19-32.

146. Fryers T, Melzer D, Jenkins R. Social inequalities and the common mental disorders: a systematic review of the evidence. *Soc Psychiatry Psychiatr Epidemiol* 2003; **38**: 229-37.

147. Galobardes B, Smith GD, Lynch JW. Systematic Review of the Influence of Childhood Socioeconomic Circumstances on Risk for Cardiovascular Disease in Adulthood. *Ann Epidemiol* 2006; **16**: 91-104.

148. Gershon AS, Dolmage TE, Stephenson A, Jackson B. Chronic obstructive pulmonary disease and socioeconomic status: a systematic review. *COPD* 2012; **9**: 216-26.

149. Goulden R, Ibrahim T, Wolfson C. Is high socioeconomic status a risk factor for multiple sclerosis? A systematic review. *Eu J Neurol* 2015; **22**: 899-911.

150. Grintsova O, Maier W, Mielck A. Inequalities in health care among patients with type 2 diabetes by individual socio-economic status (SES) and regional deprivation: a systematic literature review. *Int J Equity Health* 2014; **13**: 43.

151. Guglielmi O, Lanteri P, Garbarino S. Association between socioeconomic status, belonging to an ethnic minority and obstructive sleep apnea: a systematic review of the literature. *Sleep Med* 2019; **57**: 100-6.

152. Hanna KL, Rowe FJ. Health Inequalities Associated with Post-Stroke Visual Impairment in the United Kingdom and Ireland: A Systematic Review. *Neuroophthalmology* 2017; **41**: 117-36.

153. Houweling TA, Karim-Kos HE, Kulik MC, et al. Socioeconomic Inequalities in Neglected Tropical Diseases: A Systematic Review. *PLoS Negl Trop Dis* 2016; **10**: e0004546.

154. Karanth S, Fowler ME, Mao X, et al. Race, Socioeconomic Status, and Health-Care Access Disparities in Ovarian Cancer Treatment and Mortality: Systematic Review and Meta-Analysis. *JNCI Cancer Spectr* 2019; **3**: pkz084.

155. Kim D, Saada A. The social determinants of infant mortality and birth outcomes in Western developed nations: a cross-country systematic review. *Int J Environ Res Public Health* 2013; **10**: 2296-335.

156. Knorst JK, Sfreddo CS, de F. Meira G, Zanatta FB, Vettore MV, Ardenghi TM. Socioeconomic status and oral health-related quality of life: A systematic review and meta-analysis. *Community Dent Oral Epidemiol* 2021; **49**: 95-102.

157. Kolahdooz F, Nader F, Yi KJ, Sharma S. Understanding the social determinants of health among Indigenous Canadians: priorities for health promotion policies and actions. *Glob Health Action* 2015; **8**: 27968.

158. Lago S, Cantarero D, Rivera B, et al. Socioeconomic status, health inequalities and non-communicable diseases: a systematic review. *Journal of Public Health: From Theory to Practice* 2018; **26**: 1-14.

159. Lee CY, Im EO. Socioeconomic Disparities in Cardiovascular Health in South Korea: A Systematic Review. *J Cardiovasc Nurs* 2021; **36**: 8-22.

160. Li HO, Bailey AJ, Grose E, et al. Socioeconomic Status and Melanoma in Canada: A Systematic Review. *J Cutan Med Surg* 2021; **25**: 87-94.

161. Lindner LME, Rathmann W, Rosenbauer J. Inequalities in glycaemic control, hypoglycaemia and diabetic ketoacidosis according to socio-economic status and area-level deprivation in Type 1 diabetes mellitus: a systematic review. *Diabet Med* 2018; **35**: 12-32.

162. Liu RS, Aiello AE, Mensah FK, et al. Socioeconomic status in childhood and C reactive protein in adulthood: a systematic review and meta-analysis. *J Epidemiol Community Health* 2017; **71**: 817-26.

163. Lorant V, Deliege D, Eaton W, Robert A, Philippot P, Ansseau M. Socioeconomic inequalities in depression: a meta-analysis. *Am J Epidemiol* 2003; **157**: 98-112.

164. Lund J, Andersen AJW, Haugland SH. The social gradient in stress and depressive symptoms among adolescent girls: A systematic review and narrative synthesis. *Nor Epidemiol* 2019; **28**: 27-37.

165. Lundqvist A, Andersson E, Ahlberg I, Nilbert M, Gerdtham U. Socioeconomic inequalities in breast cancer incidence and mortality in Europe-a systematic review and meta-analysis. *Eur J Public Health* 2016; **26**: 804-13.

166. Lyle G, Hendrie GA, Hendrie D. Understanding the effects of socioeconomic status along the breast cancer continuum in Australian women: a systematic review of evidence. *Int J Equity Health* 2017; **16**: 182.

167. Mahboob A, Richmond SA, Harkins JP, Macpherson AK. Childhood unintentional injury: The impact of family income, education level, occupation status, and other measures of socioeconomic status. A systematic review. *Paediatr Child Health* 2021; **26**: e39-e45.

168. Manrique-Garcia E, Sidorchuk A, Hallqvist J, Moradi T. Socioeconomic position and incidence of acute myocardial infarction: a meta-analysis. *J Epidemiol Community Health* 2011; **65**: 301-9.

169. Mihor A, Tomsic S, Zagar T, Lokar K, Zadnik V. Socioeconomic inequalities in cancer incidence in Europe: A comprehensive review of population-based epidemiological studies. *Radiol Oncol* 2020; **54**: 1-13.

170. Morton RL, Schlackow I, Mihaylova B, Staplin ND, Gray A, Cass A. The impact of social disadvantage in moderate-to-severe chronic kidney disease: an equity-focused systematic review*. *Nephrol Dial Transplant* 2016; **31**: 46-56.

171. Muscatell KA, Brosso SN, Humphreys KL. Socioeconomic status and inflammation: a meta-analysis. *Mol Psychiatry* 2020; **25**: 2189-99.

172. O'Brien EC, Alberdi G, McAuliffe FM. The influence of socioeconomic status on gestational weight gain: a systematic review. *J Public Health* 2018; **40**: 41-55.

173. Ombrellaro KJ, Perumal N, Zeiher J, et al. Socioeconomic Correlates and Determinants of Cardiorespiratory Fitness in the General Adult Population: a Systematic Review and Meta-Analysis. *Sports Med Open* 2018; **4**: 25.

174. Pan Z, Huang M, Fang M, Xie X, Huang Z. Socioeconomic differences in hyperuricemia and gout: a systematic review and meta-analysis. *Endocrine* 2020; **69**: 286-93.

175. Parikh S, Brennan P, Boffetta P. Meta-analysis of social inequality and the risk of cervical cancer. *Int J Cancer* 2003; **105**: 687-91.

176. Petridou ET, Sergentanis TN, Perlepe C, et al. Socioeconomic disparities in survival from childhood leukemia in the United States and globally: a meta-analysis. *Ann Oncol* 2015; **26**: 589-97.

177. Peykari N, Djalalinia S, Qorbani M, Sobhani S, Farzadfar F, Larijani B. Socioeconomic inequalities and diabetes: A systematic review from Iran. *J Diabetes Metab Disord* 2015; **14**: 8.

178. Pillas D, Marmot M, Naicker K, Goldblatt P, Morrison J, Pikhart H. Social inequalities in early childhood health and development: a European-wide systematic review. *Pediatr Res* 2014; **76**: 418-24.

179. Probst C, Roerecke M, Behrendt S, Rehm J. Socioeconomic differences in alcohol-attributable mortality compared with all-cause mortality: a systematic review and meta-analysis. *Int J Epidemiol* 2014; **43**: 1314-27.

180. Quon EC, McGrath JJ. Subjective socioeconomic status and adolescent health: A meta-analysis. *Health Psychol* 2014; **33**: 433-47.

181. Read S, Grundy E, Foverskov E. Socio-economic position and subjective health and well-being among older people in Europe: a systematic narrative review. *Aging Ment Health* 2016; **20**: 529-42.

182. Schwendicke F, Dorfer CE, Schlattmann P, Foster Page L, Thomson WM, Paris S. Socioeconomic inequality and caries: a systematic review and meta-analysis. *J Dent Res* 2015; **94**: 10-8.

183. Scott A, Chambers D, Goyder E, O'Cathain A. Socioeconomic inequalities in mortality, morbidity and diabetes management for adults with type 1 diabetes: A systematic review. *PLoS ONE* 2017; **12**: e0177210.

184. Senese LC, Almeida ND, Fath AK, Smith BT, Loucks EB. Associations between childhood socioeconomic position and adulthood obesity. *Epidemiol Reviews* 2009; **31**: 21-51.

185. Sidorchuk A, Agardh EE, Aremu O, Hallqvist J, Allebeck P, Moradi T. Socioeconomic differences in lung cancer incidence: a systematic review and meta-analysis. *Cancer Causes Control* 2009; **20**: 459-71.

186. Silva VL, Cesse EA, de Albuquerque Md.e F. Social determinants of death among the elderly: a systematic literature review. *Rev Bras Epidemiol* 2014; **17**: 178-93.

187. Spencer NJ, Blackburn CM, Read JM. Disabling chronic conditions in childhood and socioeconomic disadvantage: a systematic review and meta-analyses of observational studies. *BMJ Open* 2015; **5**: e007062.

188. Tamayo T, Christian H, Rathmann W. Impact of early psychosocial factors (childhood socioeconomic factors and adversities) on future risk of type 2 diabetes, metabolic disturbances and obesity: a systematic review. *BMC Public Health* 2010; **10**: 525.

189. Tang KL, Rashid R, Godley J, Ghali WA. Association between subjective social status and cardiovascular disease and cardiovascular risk factors: a systematic review and meta-analysis. *BMJ Open* 2016; **6**: e010137.

190. Tatulashvili S, Fagherazzi G, Dow C, Cohen R, Fosse S, Bihan H. Socioeconomic inequalities and type 2 diabetes complications: A systematic review. *Diabetes Metab* 2020; **46**: 89-99.

191. Thomson K, Moffat M, Arisa O, et al. Socioeconomic inequalities and adverse pregnancy outcomes in the UK and Republic of Ireland: a systematic review and meta-analysis. *BMJ Open* 2021; **11**: e042753.

192. Uphoff E, Cabieses B, Pinart M, Valdes M, Maria Anto J, Wright J. A systematic review of socioeconomic position in relation to asthma and allergic diseases. *Eur Respir J* 2015; **46**: 364-74.

193. Uthman OA, Jadidi E, Moradi T. Socioeconomic position and incidence of gastric cancer: a systematic review and meta-analysis. *J Epidemiol Community Health* 2013; **67**: 854-60.

194. Valentin G, Pedersen SE, Christensen R, et al. Socio-economic inequalities in fragility fracture outcomes: a systematic review and meta-analysis of prognostic observational studies. *Osteoporos Int* 2020; **31**: 31-42.

195. Vart P, Gansevoort RT, Joosten MM, Bültmann U, Reijneveld SA. Socioeconomic Disparities in Chronic Kidney Disease: A Systematic Review and Meta-Analysis. *Am J Prev Med* 2015; **48**: 580-92.

196. Wagg E, Blyth FM, Cumming RG, Khalatbari-Soltani S. Socioeconomic position and healthy ageing: A systematic review of cross-sectional and longitudinal studies. *Ageing Res Rev* 2021; **69**: 101365.

197. Weightman AL, Morgan HE, Shepherd MA, Kitcher H, Roberts C, Dunstan FD. Social inequality and infant health in the UK: systematic review and meta-analyses. *BMJ Open* 2012; **2**: e000964.

198. Williams J, Allen L, Wickramasinghe K, Mikkelsen B, Roberts N, Townsend N. A systematic review of associations between non-communicable diseases and socioeconomic status within low- and lower-middle-income countries. *J Glob Health* 2018; **8**: 020409.

199. Wojcicki JM. Socioeconomic status as a risk factor for HIV infection in women in East, Central and Southern Africa: a systematic review. *J Biosoc Sci* 2005; **37**: 1-36.

200. Wu H, Meng X, Wild SH, Gasevic D, Jackson CA. Socioeconomic status and prevalence of type 2 diabetes in mainland China, Hong Kong and Taiwan: a systematic review. *J Glob Health* 2017; **7**: 011103.

201. van Zwieten A, Saglimbene V, Teixeira-Pinto A, et al. The Impact of Age on Income-Related Health Status Inequalities from Birth to Adolescence: A Systematic Review with Cross-Country Comparisons. *J Pediatr* 2018; **203**: 380-90.
